# Novel Synthetic Derivative of Renieramycin T Right-Half Analog Induces Apoptosis and Inhibits Cancer Stem Cells via Targeting the Akt Signal in Lung Cancer Cells

**DOI:** 10.3390/ijms24065345

**Published:** 2023-03-10

**Authors:** Korrakod Petsri, Masashi Yokoya, Satapat Racha, Sunisa Thongsom, Chorpaka Thepthanee, Bhurichaya Innets, Zin Zin Ei, Daiki Hotta, Hongbin Zou, Pithi Chanvorachote

**Affiliations:** 1 Center of Excellence in Cancer Cell and Molecular Biology, Faculty of Pharmaceutical Sciences, Chulalongkorn University, Bangkok 10330, Thailand; 2 Department of Pharmacology and Physiology, Faculty of Pharmaceutical Sciences, Chulalongkorn University, Bangkok 10330, Thailand; 3 Department of Pharmaceutical Chemistry, Meiji Pharmaceutical University, 2-522-1, Noshio, Kiyose, Tokyo 204-8588, Japan; 4 Interdisciplinary Program in Pharmacology, Graduate School, Chulalongkorn University, Bangkok 10330, Thailand; 5 College of Pharmaceutical Sciences, Zhejiang University, Hangzhou 310058, China

**Keywords:** derivatives of renieramycin T right-half analog, lung cancer, apoptosis, Akt, cancer stem cells, structure-activity relationships

## Abstract

Akt is a key regulatory protein of cancer stem cells (CSCs) and is responsible for cancer aggressiveness and metastasis. Targeting Akt is beneficial for the development of cancer drugs. renieramycin T (RT) has been reported to have Mcl-1 targeting activity, and the study of the structure-activity relationships (SARs) demonstrated that cyanide and the benzene ring are essential for its effects. In this study, novel derivatives of the RT right-half analog with cyanide and the modified ring were synthesized to further investigate the SARs for improving the anticancer effects of RT analogs and evaluate CSC-suppressing activity through Akt inhibition. Among the five derivatives, a compound with a substituted thiazole structure (DH_25) exerts the most potent anticancer activity in lung cancer cells. It has the ability to induce apoptosis, which is accompanied by an increase in PARP cleavage, a decrease in Bcl-2, and a diminishment of Mcl-1, suggesting that residual Mcl-1 inhibitory effects exist even after modifying the benzene ring to thiazole. Furthermore, DH_25 is found to induce CSC death, as well as a decrease in CSC marker CD133, CSC transcription factor Nanog, and CSC-related oncoprotein c-Myc. Notably, an upstream member of these proteins, Akt and p-Akt, are also downregulated, indicating that Akt can be a potential target of action. Computational molecular docking showing a high-affinity interaction between DH_25 and an Akt at the allosteric binding site supports that DH_25 can bind and inhibit Akt. This study has revealed a novel SAR and CSC inhibitory effect of DH_25 via Akt inhibition, which may encourage further development of RT compounds for cancer therapy.

## 1. Introduction

Lung cancer is the most prevalent cause of cancer death, and according to estimates of the global cancer statistics of incidence and mortality of all types of malignancies, its incidence is increasing. Furthermore, the 5-year survival rate of lung cancer is extremely low as a result of the disease’s recurrence and resistance to cancer treatment [1,2]. The use of more precise medications aimed at the molecular targets underlying cancer aggressiveness and its drug resistance has been suggested to be useful by the increased clinical response driven by targeted therapy [3,4].

Recently, the Akt signaling pathway has been considered a promising therapeutic target in cancer therapy. Several drugs that target Akt, such as ipatasertib and capivasertib, are being evaluated in clinical trials [5]. According to studies, Akt signaling is often upregulated, leading to increased proliferation, survival, and resistance to chemotherapy. Phosphorylated Akt was related to a poor prognosis in patients with non-small cell lung cancer (NSCLC) [6]. The overactivation of the key molecules in this pathway enhances cancer cell survival by inhibiting cell death [7]. Importantly, Akt has been shown to play a critical role in the maintenance of cancer stem cells (CSCs) [8], which are a small subpopulation of cancer cells that exhibit stem cell-like characteristics, such as self-renewal and the capacity to develop into several cell types. CSCs have been involved in the development, metastasis, and resistance to cancer treatment [9]. CSCs with high tumorigenic potential is dependent on certain stem cell-related signaling pathways such as Akt signaling, Wnt signaling, Notch signaling, and Hedgehog signaling [10,11]. However, the Akt signaling pathway seems to be the most crucial pathway in CSCs [8]. It has been reported that Akt itself and the downstream signal of Akt, especially c-Myc, maintain cancer stemness through transcriptional activation of the CSC-associated genes [12,13,14]. It was demonstrated that c-Myc downregulation inhibited the capacity of colon CSCs for self-renewal and xenograft development [15]. Therefore, self-renewal and chemoresistance of CSCs are critically dependent on the Akt signaling pathway, and the compounds that could target Akt are good candidates for therapeutic intervention.

Renieramycin T (RT), a tetrahydroisoquinoline alkaloid from the renieramycin group that is isolated from the blue sponge *Xestospongia* sp., was found to be primarily cytotoxic for lung cancer cells and to cause apoptosis by targeting Mcl-1 for ubiquitin-proteasomal degradation [16]. It has been reported that RT derivatives have activities on CSCs and the Akt signal. 5-*O*-acetyl-renieramycin T was demonstrated to eliminate lung CSCs and make lung cancer cells more susceptible to cisplatin-mediated apoptosis [17]. Moreover, 5-*O*-(*N*-Boc-l-alanine)-renieramycin T (OBA-RT) showed a lung CSC-suppressing effect through Akt inhibition [18].

Despite its anticancer properties, RT’s complex and large structure makes large-scale synthesizing difficult. To resolve this issue, previous studies used reinieramycins as a lead compound to synthesize the right- and left-halves of reinieramaycins, aiming to reduce the complexity of their structures and investigate their anticancer activities. Results revealed that the right-half RT compounds exhibited stronger anti-cancer activity than the left-half in cancer cells [19,20]. Consequently, five right-half RT analogs were synthesized to study their structure–activity relationships (SARs) and identify active moieties that are essential for drug action. Results of the study indicated that the cyanide and benzene ring compositions of RT play an important role in targeting Mcl-1 [21]. Based on these findings, novel synthetic derivatives of RT right-half analogs were developed by modifying the other part of structure, including the ring part, with the remaining cyanide to explore the SARs and improve the anticancer effects. Moreover, the activity on CSCs of the most effective derivative of RT right-half analog (DH_25) was also evaluated to investigate its CSC-suppressing effects through Akt signal inhibition. As CSCs help cancer cells to survive, these findings support the innovation of cancer treatments and the ongoing development of RT derivatives as a new compound that not only targets Mcl-1 but also inhibits CSCs by Akt suppression.

## 2. Results

### 2.1. Derivatives of RT Right-Half Analog of RT Induce Cytotoxicity, Apoptosis, and Morphological Changes in NSCLC Cells

**Figure 1:**
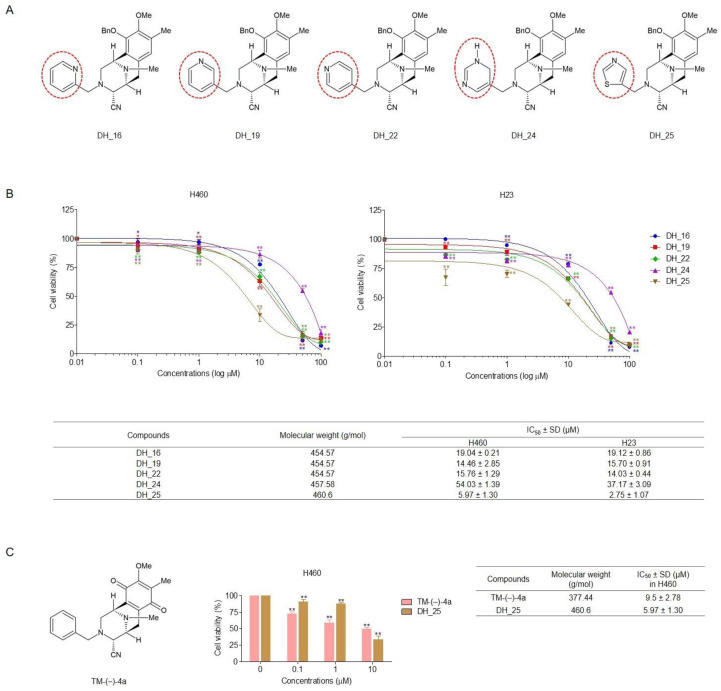
Derivatives of RT right-half analogs (DH_16, DH_19, DH_22, DH_24, DH_25, and TM-(–)-4a) induce cytotoxicity in NSCLC cells (H460 and H23). (**A**) Structures of DH_16, DH_19, DH_22, DH_24, and DH_25. (**B**) NSCLC cells were seeded and treated with 0–100 µM of DH_16, DH_19, DH_22, DH_24, and DH_25 for 24 h. Then, the MTT assay was performed to evaluate cell viability. IC_50_ was calculated in comparison with the untreated control. (**C**) Structures and the cytotoxicity profile of TM-(–)-4a in H460 cells. The IC50 was calculated in comparison with the untreated control. Data are represented as the mean ± SD (*n* = 3) (* 0.01 ≤ *p* < 0.05, ** *p* < 0.01, compared with the untreated control).

To investigate the SARs and identify the most effective compound for further experiments, the cytotoxic profile was first evaluated using an MTT assay. Derivatives of the RT right-half analog containing a different modified ring in their structures (Figure 1A) were used to treat NSCLC cells (H460 and H23) with several concentrations for 24 h, and then cell viability was analyzed. The results showed that all compounds significantly exerted cytotoxic effects with half-maximal inhibitory concentrations (IC_50_) lower than 60 µM (Figure 1B) compared with the untreated control. DH_25 was the most effective compound in both NSCLC cells (H460 and H23) with IC_50_ of 5.97 ± 1.30 and 2.75 ± 1.07 µM, respectively, whereas DH_24 had the lowest anticancer effect with IC_50_ of 54.03 ± 1.39 and 37.17 ± 3.09 µM, respectively. The IC_50_ values of DH_16, DH_19, and DH_22 were comparable at 14–20 µM. Moreover, when TM-(–)-4a, the most potent compound in a previous study [21], was applied for comparison, DH_25 even showed a lower IC_50_ than TM-(–)-4a, whose IC_50_ in H460 cells was 9.5 ± 2.78 µM (Figure 1C).

**Figure 2:**
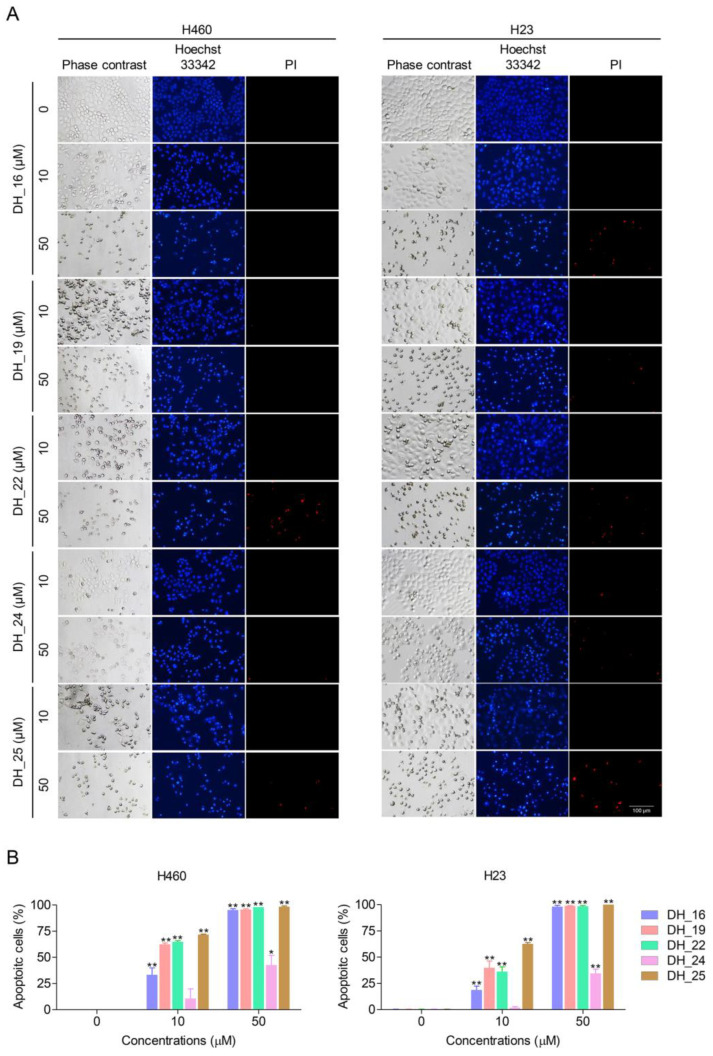
Derivatives of the RT right-half analog (DH_16, DH_19, DH_22, DH_24, and DH_25) induce apoptotic morphological changes in NSCLC cells (H460 and H23). (**A**,**B**) NSCLC cells were seeded and treated with 0–50 µM of DH_16, DH_19, DH_22, DH_24, and DH_25 for 24 h. Hoechst 33342 and PI were used to stain the cell nuclei. Images were obtained under a fluorescence microscope, and the percentages of apoptotic cells were calculated. Data are presented as the mean ± SD (*n* = 3) (* 0.01 ≤ *p* < 0.05, ** *p* < 0.01, compared with the untreated control).

Apoptosis is an important mode of cell death induced by most anticancer drugs. During the apoptosis process, morphological changes including shrinkage of nuclei, cytoplasmic shrinkage, nuclear chromatin condensation, dilated endoplasmic reticulum, and membrane blebbing were detected. To evaluate the mode of cell death in response to derivatives of RT right-half analog treatment, a nuclear staining assay was performed. A red fluorescence of PI indicated a necrotic cell; however, a bright blue fluorescence of Hoechst 33342 clearly detected in the representation of condensed and fragmented chromatin indicated an early stage of apoptosis. Condensed and/or fragmented nuclei were seen after 24 h of treatment, demonstrating significantly increased apoptosis of NSCLC cells when treated with 10–50 µM of DH_16, DH_19, DH_22, and DH_25 and 50 µM of DH_24 compared with the untreated control (Figure 2A,B). According to a previous experiment, DH_25 with the benzene ring replaced by a thiazole structure had a potent cytotoxicity effect; this experiment also demonstrated DH_25’s most effective apoptosis-induction effects. DH_25 caused 60–70% of dead cells in NSCLC cells at 10 µM, which is much more than the other compounds (Figure 2A,B). Therefore, DH_25 was selected in the next experiment.

### 2.2. DH_25 Inhibits Colony-Forming Activity and Induces Apoptosis in NSCLC Cells

**Figure 3:**
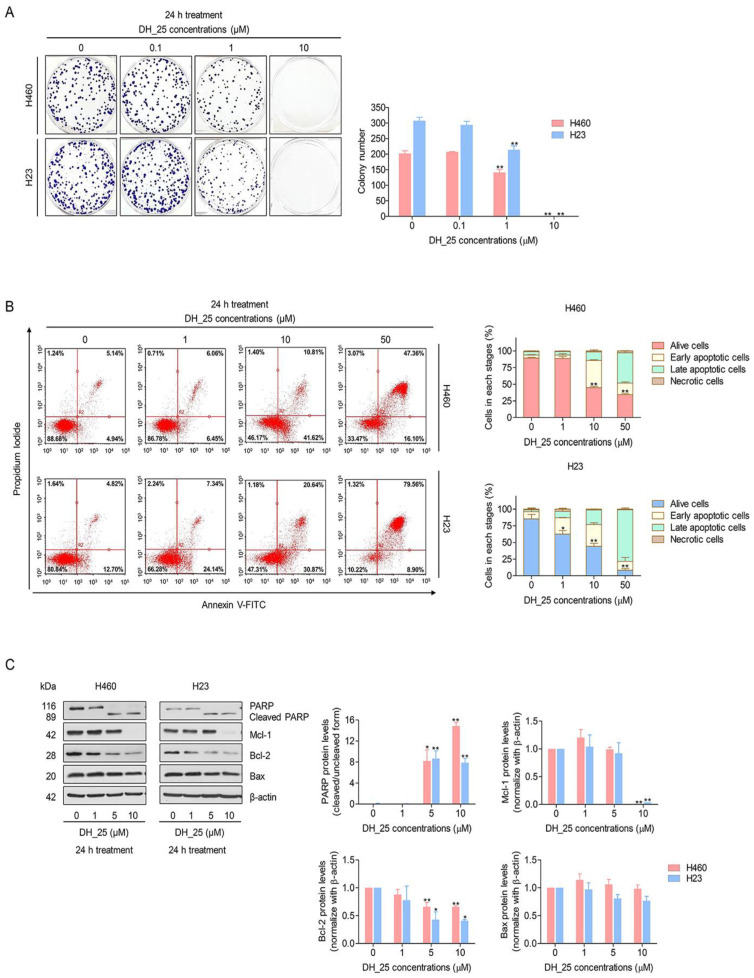
DH-25 inhibits colony formation and induces apoptosis in NSCLC cells (H460 and H23). (**A**) NSCLC cells were seeded and treated with 0–10 µM of DH_25 for 24 h. The cells were then cultured for seven days before being stained with crystal violet to count the colony number. (**B**) NSCLC cells (H460 and H23) were seeded and treated with 0–50 µM of DH_25 for 24 h. Flow cytometry was used to detect apoptotic cells using annexin V-FITC/PI staining. The percentages of cells in each stage were calculated. (**C**) NSCLC cells (H460 and H23) were seeded and treated with 0–10 µM of DH_25 for 24 h. Western blot analysis was performed to detect the protein levels of PARP, cleaved PARP, Mcl-1, Bcl-2, and Bax. The blots were reprobed with β-actin to confirm an equal loading. Densitometry was used to calculate protein expression levels, and results were presented as fold changes relative to the uncleaved form, or β-actin. The uncropped blotting bands are presented in Figure S6. Data are presented as the mean ± SD (*n* = 3) (* 0.01 ≤ *p* < 0.05, ** *p* < 0.01, compared with the untreated control).

The colony formation assay, a cell proliferation assay that can identify a single cell’s capacity to develop into a colony, was utilized to verify the anticancer action of DH_25. After 7 days of DH_25 treatment, the crystal violet-staining colony represented the colony suppression effect of DH_25 compared with the untreated control (Figure 3A). The results revealed that DH_25 significantly reduced the colony number from 202 to 141 colonies in H460 cells and from 307 to 214 colonies in H23 cells. Moreover, with the treatment of 10 µM of DH_25, colonies were completely abolished, indicating the strong effects of DH_25 (Figure 3A).

Since apoptotic morphological changes were previously screened by Hoechst 33342 and PI double staining, flow cytometric analysis using annexin V-FITC/PI staining was applied to validate the apoptosis induced by DH_25. Similar to the nuclear staining assay, DH_25 induced apoptosis, starting at a concentration of 0–10 µM in both NSCLC cells (Figure 3B). To confirm the apoptosis mechanism, Western blot analysis was further utilized to investigate apoptosis-related proteins such as PARP, Mcl-1, Bcl-2, and Bax. The results revealed an increase in cleaved PARP in response to DH_25 (5–10 μM) treatment compared with control. Bcl-2 protein was found to significantly decrease at 5–10 μM of DH_25 treatment, whereas no effect on Bax protein levels was found in both NSCLC cells (Figure 3C). Consistently, the protein analysis of Mcl-1 indicated that the treatment of cells with DH_25 diminished the Mcl-1 protein in both NSCLC cells at a concentration of 10 μM, confirming a DH_25 remaining Mcl-1-targeting effect (Figure 3C). Taken together, these results suggest that DH_25’s apoptosis mechanism involved Mcl-1 protein targeting and, at least in part, Bcl-2 suppression. The modification of the benzene ring structure did not affect the Mcl-1-targeting mechanism.

### 2.3. DH_25 Suppresses CSC Spheroid Formation and CSC Signals in NSCLC Cells

**Figure 4:**
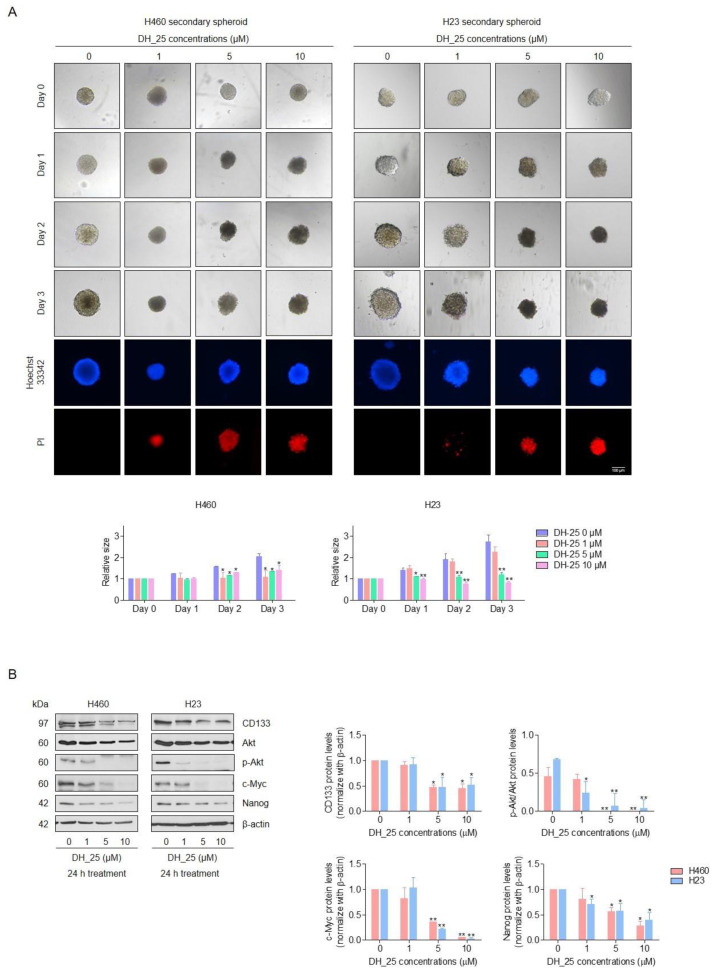
DH-25 suppresses CSC spheroid formation and CSC signals in NSCLC cells (H460 and H23). (**A**) NSCLC cells (H460 and H23) were seeded and allowed to form primary spheroids for 7 days. The primary spheroids were suspended into single cells to form CSC-rich spheroids for 14 days in ultralow-attachment 96-well plates. The CSC-rich spheroids were then treated with 0–10 µM of DH_25 for 3 days. Hoechst 33342 and PI were used to stain the cell nuclei. Images were visualized using a fluorescence microscope. The relative size of CSC spheroids was quantified. (**B**) NSCLC cells (H460 and H23) were seeded and treated with 0–10 µM of DH_25 for 24 h. Western blot analysis was performed to detect the protein levels of CD133, Akt, p-Akt, c-Myc, and Nanog. The blots were reprobed with β-actin to confirm an equal loading. Densitometry was used to calculate protein expression levels, and results were presented as fold changes relative to the uncleaved form, or β-actin. Uncropped blotting bands are presented in Figure S7. Data are presented as the mean ± SD (*n* = 3) (* 0.01 ≤ *p* < 0.05, ** *p* < 0.01, compared with the untreated control).

CSCs have emerged as a crucial target for the development of new anticancer agents. Then, the activity of DH_25 to suppress CSC-like phenotypes and the apoptotic induction of DH_25 in the CSC population were evaluated by the three-dimensional (3D) spheroid formation assay, followed by Hoechst 33342 and PI double staining. After 3 days of DH_25 treatment on CSC-rich cancer cells, which were isolated and grown in 96-well plates, the treatment of CSC-rich populations with 5–10 µM of DH_25 caused a reduction in CSC survival in both cells, with a significant decrease in the relative size of the H460 and H23 CSC spheres compared with the untreated control since days 1–3 after treatment (Figure 4A). In addition, the DH_25-treated spheroids were found to detach and dissociate, whereas the untreated spheroids showed normal survival features (Figure 4A). The apoptotic character of DNA fragmentation and/or DNA condensation in DH_25-treated spheroids was further demonstrated by Hoechst 33342 and PI double staining on day three of treatment (Figure 4A). Gathering from these data, we found that DH_25 demonstrated anti-CSC characteristics that might cause CSC apoptosis.

The clinical prognosis of CSC-driven cancers, such as lung cancer, may be decreased and improved by inhibiting cellular signals that maintain CSCs. Several pathways control the stemness of cancer, and the Akt pathway can control Nanog, a pluripotent transcription factor. Akt inhibition can lead to depletion of the stem cell transcription factor, resulting in a reduction of CSC phenotypes. To confirm the CSC-suppressing effects and investigate the upstream regulatory signals of CSC in response to DH_25 treatment, the expression of a CSC marker (CD133), CSC transcription factor (Nanog), and CSC key signaling proteins, including Akt, p-Akt, and c-Myc, were determined by Western blot analysis. After DH_25 treatment, the protein levels of CD133 and Nanog were downregulated in both NSCLC cells (Figure 4B). Likewise, Akt, p-Akt, and c-Myc, which are known as the upstream CSC regulatory proteins, decreased in response to DH_25 treatment (Figure 4B). As p-Akt is the active form of Akt, a decrease in the p-Akt/Akt ratio means that the protein is inactive after the treatment with DH_25. These results suggest that DH_25 might have an ability to target the upstream CSC regulatory protein, Akt.

### 2.4. DH_25 Directly Interacts with Akt, the Upstream Regulatory Signal of CSC

**Figure 5:**
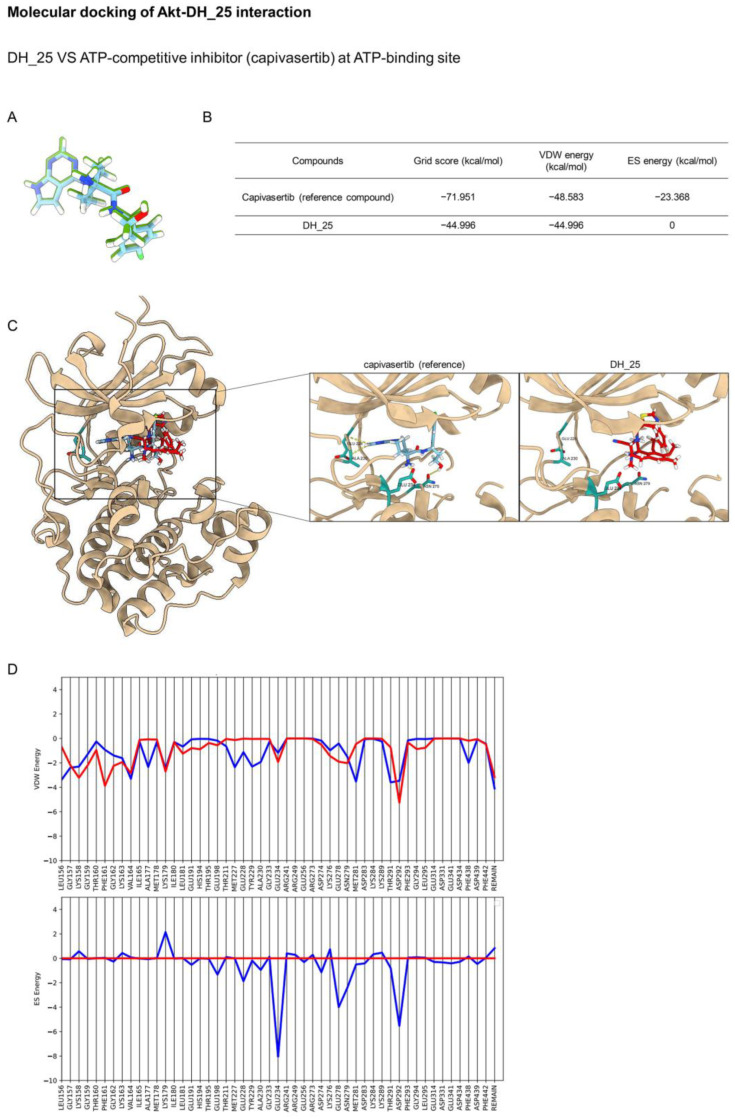
Molecular docking of DH_25 and Akt at the ATP-binding site compared with the ATP-competitive inhibitor (capivasertib). (**A**) Structural superimposition of redocked (blue) and experimental native ligands (green) at the active site of the ATP-binding pocket. (**B**) Binding energies of the ligand in complex with the ATP-binding site of Akt. (**C**) The ATP-binding site of Akt complexed with DH_25 or capivasertib, for reference. The yellow dashed lines denote hydrogen-bonding interactions. (**D**) Footprint analysis for DH_25 (red lines) compared with the capivasertib reference (blue lines) into the ATP-binding site of Akt.

**Figure 6:**
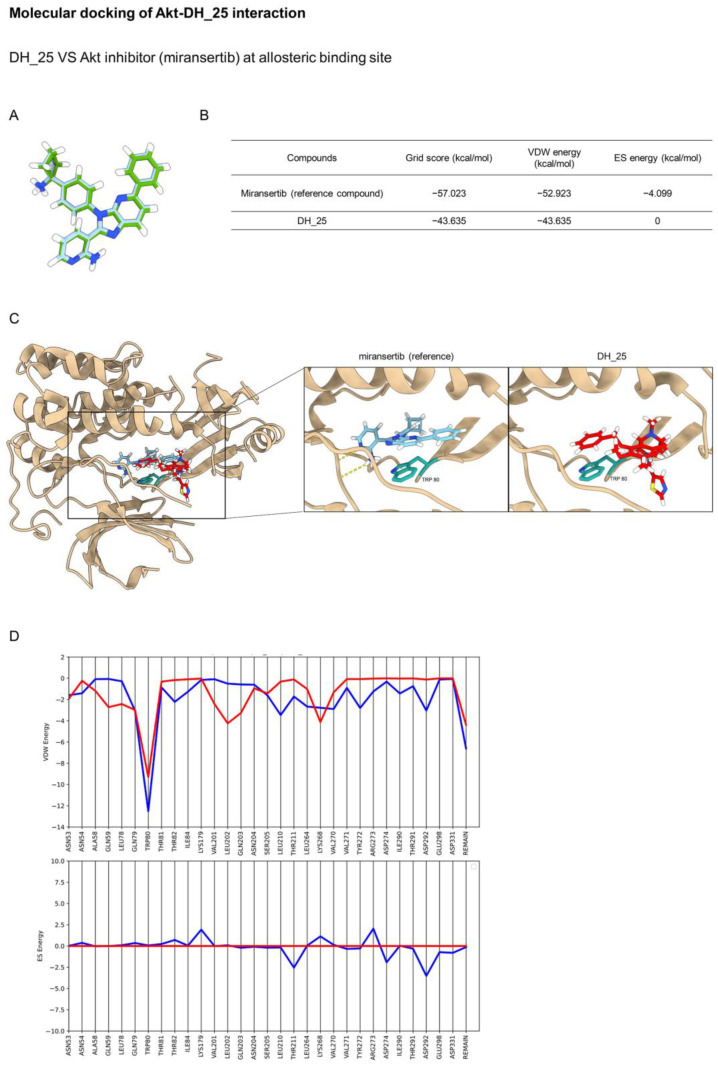
Molecular docking of DH_25 and Akt at the allosteric binding site in comparison to miransertib, an Akt inhibitor. (**A**) Structural superimposition of redocked (blue) and experimental native ligands (green) at the allosteric binding site. (**B**) Binding energies of the ligand in complex with the allosteric binding site of Akt. (**C**) The allosteric binding site of Akt in complex with DH_25 or miransertib for reference. The yellow dashed lines denote hydrogen-bonding interactions. (**D**) Footprint analysis for DH_25 (red lines) compared with the miransertib reference (blue lines) into the allosteric binding site of Akt.

**Figure 7:**
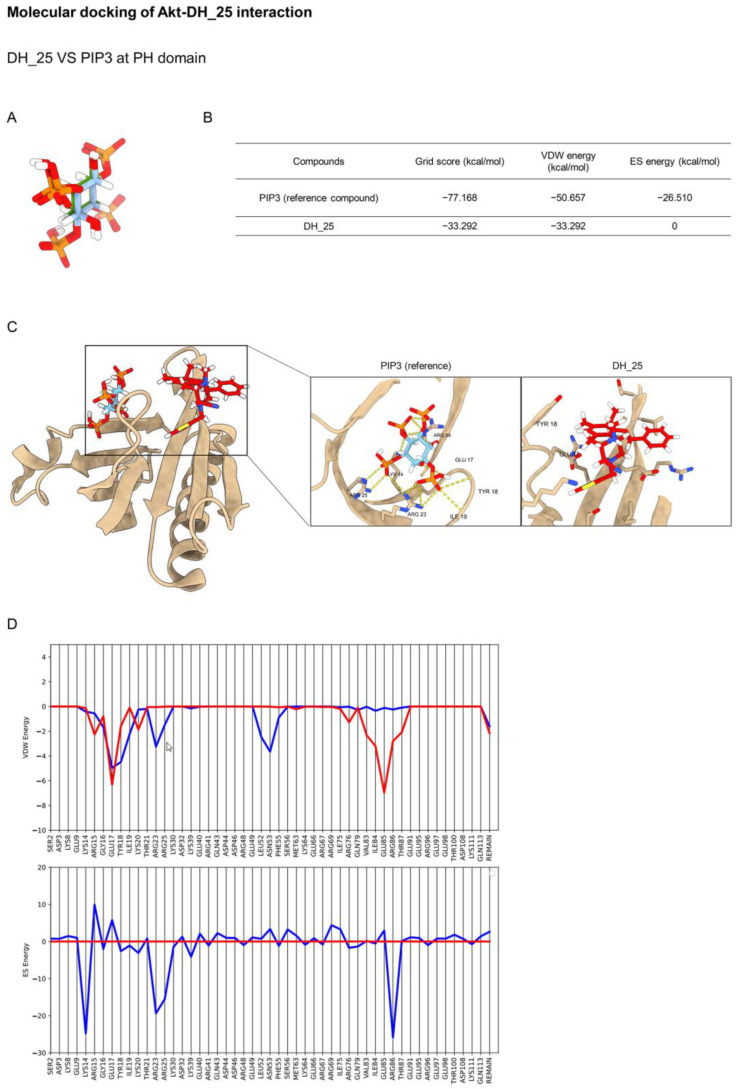
Molecular docking of DH_25 and Akt at the PH domain compared with PIP3. (**A**) Structural superimposition of redocked (blue) and experimental native ligand (green) at the PH domain. (**B**) Binding energies of the ligand in complex with the PH domain of Akt. (**C**) The PH domain of Akt in complex with DH_25 or PIP3 for reference. The yellow dashed lines denote hydrogen-bonding interactions. (**D**) Footprint analysis for DH_25 (red lines) compared with the PIP3 reference (blue lines) into the PH domain of Akt.

Akt has been reported as the main regulatory key of CSC [8], and this protein is the upstream signal regulating c-Myc [22]. The three main classes of Akt inhibitors were investigated and entered into preclinical and clinical trials, namely, ATP-competitive, allosteric, and pleckstrin homology (PH) domain inhibitors [23]. Regarding the previous experiment, molecular docking simulation was applied to investigate the interaction between the DH_25 compound and Akt protein using the co-crystal structures of an ATP-competitive inhibitor (PDB ID 4GV1), an allosteric inhibitor (PDB ID 5KCV), and a PH domain (PDB ID 5KCV). Initially, the Dock6.9 protocol was validated by re-docking the original co-crystallized ligands. The calculated root mean square deviations between the experimental and docked conformations are 0.8881 Å, 0.1876 Å, and 0.4669 Å, which confirms that the docking protocol used in this investigation is suitable for the prediction (Figure 5A, Figure 6A and Figure 7A).

ATP-competitive inhibitors belong to the largest group of Akt inhibitors. On the opened PH-out conformation of Akt, these molecules occupy the same site with adenosine triphosphate (ATP). Capivasertib (AZD5363) is a clinical candidate that targets Akt’s selective ATP-binding pocket. Based on the molecular docking study, the binding energies of DH_25 and the co-crystal ligand capivasertib are presented in Figure 5B. Capivasertib was found to interact with Leu156, Val164, Ala177, Lys179, Met227, Glu228, Tyr229, Ala230, Glu234, Glu278, and Asp292 (−71.951 kcal/mol). On the contrary, DH_25 had binding energies (−44.996 kcal/mol) and did not form a hydrogen bond with residues Glu228, Ala230, Glu234, and Asn279 (Figure 5C). This could suggest that ES energy showed the most significant contribution to the interaction between the ligands and residues at the ATP-binding site. Footprint analysis showed that DH_25 has less favorable VDW energies than capivasertib at specific residues such as Val164, Met227, Tyr229, Met281, and Thr291, and a slightly unfavorable ES energy with Glu234 (Figure 5D). The findings demonstrated that DH_25 did not significantly bind to the ATP-binding pocket.

Miransertib (ARQ 092) is an orally available, selective Akt inhibitor, that was employed as a reference molecule in the docking method. The docking results of DH_25 and miransertib with their allosteric binding sites are presented in Figure 6B. Miransertib binds with Akt at a unique interdomain region between the kinase and PH domains and stabilizes Akt with the key residue Trp80. DH_25 adopted a similar binding mode to miransertib, with an interface between the kinase and PH domains (Figure 6C). This could suggest that VDW energy showed the most significant contribution to the interaction between the ligands and residues at the allosteric binding site. According to the footprint analysis, DH_25 inhibited Akt with Trp80 (−9.287 kcal/mol) to fit with the allosteric binding site (Figure 6D). Thus, unlike ATP-competitive inhibitors, allosteric inhibitors degrade enzyme phosphorylation [24]. This result confirmed that DH_25 significantly inhibited Akt via an allosteric site.

The detailed structure of DH_25 in the N-terminal PH domain of Akt is shown in Figure 7C. This class of Akt inhibitors can attach to the phosphoinositide-binding pocket of the Akt PH domain, preventing PIP3 from interacting with the inner membrane. DH_25 was located in different parts of the PH domain. On the contrary, DH_25 showed less good interaction (Figure 7B) because it has a larger grid score (−33.292 kcal/mol) than the reference compound (−77.168 kcal/mol). Moreover, footprint analysis illustrated that DH_25 could not mimic the interaction patterns made by the reference compound (Figure 7D). The findings demonstrated that DH_25 did not significantly bind to the PH domain.

Even the docking results showed the effective binding between DH_25 and the allosteric site of Akt, which was according to the Western blotting results (Figure 4B), but there is still a question about whether DH_25 could interact with the key proteins in the other CSC regulatory pathways, including Wnt, Notch, and Hedgehog, or not. Therefore, molecular docking was performed to investigate this point. The results are presented in Supplemental Information 1, Figures S1 and S2. It was demonstrated that DH_25 has limited or no activity as an inhibitor of the Wnt, Notch, and Hedgehog pathways (Figures S1 and S2).

**Figure 8:**
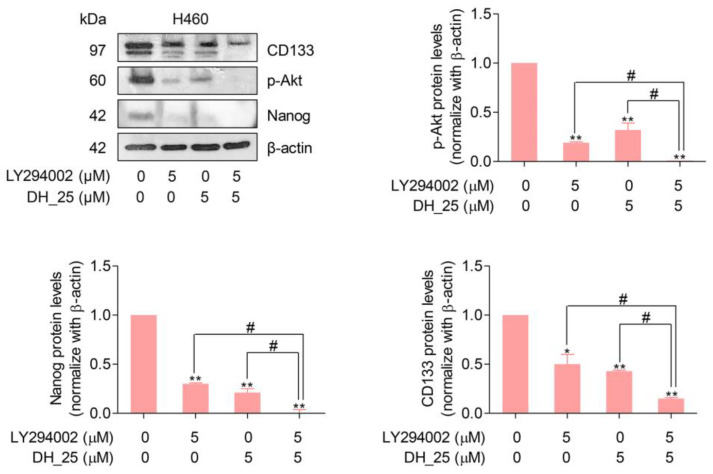
DH-25 regulates Nanog and CD133 through p-Akt inhibition in NSCLC cells (H460). NSCLC cells (H460) were seeded and treated with 0–10 µM of DH_25 for 24 h. In the conditions that LY294002 (5 µM) was used, it was added to pretreat the cells for 0.5 h before treatment with DH_25. Western blot analysis was performed to detect the protein levels of p-Akt, Nanog, and CD133. The blots were reprobed with β-actin to confirm an equal loading. Densitometry was used to calculate protein expression levels, and results were presented as fold changes relative to the uncleaved form, or β-actin. Uncropped blotting bands are presented in Figure S8. Data are presented as the mean ± SD (*n* = 3) (* 0.01 ≤ *p* < 0.05, ** *p* < 0.01, compared with the untreated control, and # 0.01 ≤ *p* < 0.05, compared with the treatment alone).

Based on the findings that DH_25 directly targeted the Akt protein (Figure 6), the absence of Akt signaling led to the inactivation of its downstream signals, resulting in a decrease in CSC markers, CD133 and Nanog. To confirm that the downstream CSC markers, Nanog and CD133, were regulated by Akt and affected by DH_25 treatment through its Akt targeting activity, the Western blotting experiment with a PI3K/Akt pathway inhibitor, LY294002 (2-4-morpholinyl-8-phenlchromone), was performed. Inactivation of PI3K using LY294002 has been shown to lead to dephosphorylation of Akt at Thr308 and Ser473, consequently inducing cell apoptosis [25]. After the treatments, the results demonstrated that when treated with the combination of DH_25 (5 µM) and LY294002 (5 µM), the protein levels of CD133 and Nanog were decreased more than with the treatment of each compound alone (Figure 8). These results were correlated with their upstream signaling, p-Akt. The protein levels of p-Akt were found to be completely diminished in response to the combination treatment, whereas they were still found at decreased levels with the treatment alone (Figure 8). Gathering from the same trending results, it could be suggested that CD133 and Nanog were regulated by the Akt signal.

## 3. Discussion

Renieramycins are one of the natural marine-derived products. They are a member of the tetrahydroisoquinoline family of alkaloids, which is isolated from various marine species, including the genera *Reniera* [26], *Xestospongia* [27,28], *Cribrochalina* [29], and *Neopetrosia* [30] of sponges. Recently, renieramycins have shown a potent ability as anticancer agents [31,32,33,34], especially renieramycin T (RT), which was successfully isolated from the blue sponge *Xestospongia* sp. in 2009 [35]. RT has anti-cancer activity against including colon, prostate [36], NSCLC [37], breast (T47D), and pancreatic (AsPC1) cancer cells [35]. Furthermore, a modified form of RT named 5-*O*-acetyl-renieramycin T eliminates lung CSCs and makes lung cancer cells more susceptible to cisplatin-mediated apoptosis [17]. 5-*O*-(*N*-Boc-l-alanine)-renieramycin T, another RT-modified compound, also binds and inhibits Akt function, resulting in apoptosis and cancer stem cell suppression [18]. A previous study of RT showed that this compound exerts apoptosis-induction effects by directly targeting Mcl-1, a key anti-apoptotic protein, and inducing its degradation through ubiquitin-proteasomal degradation [16].

Despite the remarkable anticancer properties of RT and its modified forms, it cannot be denied that they are difficult to synthesize in large-scale productions because of their complex and large-scale structures. Therefore, information about its SARs is required. As there is a correlation between molecular structures and biological activity, SARs can be used to predict the activities of new molecules from their molecular structures, which is useful information for further anticancer drug modification and drug development [38]. SARs are essential to many aspects of new drug discovery. This enables bioactive compound’s effect or potency to be modulated by modifying its chemical structure.

As part of the projects investigating renieramycin T (RT) and its derivatives for anticancer activities, model compounds in both the right- and left-halves of the renieramycins have been previously synthesized and studied [19,20]. The studies have shown that the right-half RT compounds are more suitable for further development based on their anti-cancer activity. According to studies by Nakai K et al. and Matsubara T et al. [19,20], the most effective left-half compound, 10a, has an IC_50_ of 0.054 µM in colorectal cancer cells (HCT116) and 0.22 µM in lung cancer cells (QG56), while other left-half compounds have an IC_50_ of more than 0.1 µM in both cells after 4 days of compound treatments. In contrast, three of the right-half compounds (1m, (±)-6a, and (–)-6a) have much lower IC_50_ values than the left-half compounds, which are (18.1 ± 1.4) × 10^−3^, (11.4 ± 1.0) × 10^−3^, and (12.5 ± 0.5) × 10^−3^ µM, respectively, in HCT116 cells and (4.0 ± 0.9) × 10^−3^, (14.0 ± 0.6) × 10^−3^, and (11.9 ± 2.2) × 10^−3^ µM, respectively, in QC56 cells. In summary, the cytotoxicity effects of the right-half compounds are superior to those of the left-half compounds. Therefore, the right half of renieramycin T has been chosen for further modification.

Cyanide and a benzene ring of the RT analog are necessary components for the interaction with Mcl-1, as indicated by further studies of SARs [21]. However, the IC_50_ of the Mcl-1-targeted RT right-half analogs was quite high compared with that of the parent compound. Consequently, this study was conducted to further investigate the SARs and improve the anticancer effects of RT right-half analogs. Five novel derivatives of the RT right-half analog were synthesized with the remaining cyanide and ring, which have been previously reported as essential structures for Mcl-1-targeting activity. The benzene ring from the parent compound was modified by changing the carbon molecule to a nitrogen molecule, i.e., pyridine (DH_16, DH_19, and DH_22) and dihydropyrimidine (DH_24), and replacing the benzene with thiazole (DH_25) (Figure 1A). After treating the NSCLC cells with all compounds, the cytotoxicity profiles and apoptosis characteristics were determined. DH_16, DH_19, and DH_22 demonstrated comparable cytotoxicity and apoptotic cell percentages, indicating that changing the nitrogen position within the pyridine structure had no effect on their activities (Figure 1B and Figure 2). DH_24 showed the highest IC_50_ with the lowest apoptotic cell percentages among compounds (Figure 1B and Figure 2), which might be related to its very large dihydropyrimidine structure. Conversely, DH_25, a thiazole-based compound, demonstrated the most potent activities, as evidenced by the lowest IC_50_ and the highest apoptotic cell induction by DH_25 at 10 µM (Figure 1B and Figure 2). In addition, this compound even showed more effects than TM-(–)-4a, which was the most potent analog in a previous study (Figure 1C). Furthermore, it demonstrated the inhibitory effect of colony formation in NSCLC cells at a low concentration (1 µM), supporting its high anticancer potency (Figure 3A). Thiazole was reported as a good pharmacophore nucleus on the basis of its pharmaceutical applications. The compounds containing thiazole show a wide range of biological activities, such as antioxidant, analgesic, antimicrobial, anti-inflammatory, and anticancer activities. More than 18 FDA-approved drugs and several developmental drugs contain the thiazole scaffold [39]. This might be the reason why DH_25 could exert the best anticancer activity among derivatives of the RT right-half analog. Therefore, DH_25 was chosen for further investigation of other mechanisms.

In cancer biology, the evasion of apoptosis is a well-known hallmark of cancer. As it helps eliminate unwanted cells and preserves the balance between cell survival and cell death, apoptosis is a crucial part of many processes [40]. As dysregulated apoptosis leads to persistent cell proliferation and accelerated tumor growth, drugs or therapeutic approaches that can reactivate apoptotic signaling pathways may be beneficial for the treatment of cancer. Having shown the apoptosis morphology in response to DH_25 treatment, the apoptosis-induction effect of DH_25 was clarified by annexin V-FITC/PI staining, and its mechanism was confirmed by Western blot analysis (Figure 3B,C). The increase in apoptotic cells in the early and late stages (Figure 3B) and the cleaved PARP protein levels proved the apoptosis-induction activities of DH_25 (Figure 3C). In addition, the investigation of the apoptotic-related proteins also revealed that DH_25 diminished the expression of the anti-apoptotic protein Mcl-1 and partly decreased Bcl-2 levels (Figure 3C). This finding adds to previous research that both the cyanide and benzene rings of RT analogs are essential for Mcl-1-targeting activity [21]. This study suggests that even if the benzene ring is modified to thiazole, the Mcl-1-suppression effect is preserved. Thus, the key moieties of RT analogs that led to the Mcl-1 depletion mechanism were cyanide and the ring part (benzene or thiazole).

A subpopulation of cancer cells known as CSCs exhibit stemness features, such as the capacity to divide asymmetrically. Due to their self-renewal ability, CSCs produce a heterogeneous population of cancer cells. As a result, CSCs are considered notably more tumorigenic than other cancer cells and responsible for the biological traits of cancer such as rapid growth, invasion, and metastasis [41,42]. Hence, CSCs have been proposed as potential targets or drug receptors in cancer treatment. Indeed, various anti-CSC strategies have been investigated by inhibiting numerous intracellular signaling pathways, including Wnt, Notch, Hedgehog, and Akt [11]. Akt signaling is believed to be a key regulator of CSC phenotypes and properties [43]. Previous studies have revealed that Akt is closely related to Nanog, the major transcription factor, and c-Myc, an oncoprotein that regulates CSC self-renewal and pluripotency and acts as a prognostic indicator in lung CSCs [44]. Several studies have supported the view that Akt inhibition would lead to the suppression of CSCs. GSK690693, an Akt inhibitor currently in phase I clinical trials, was reported to inhibit cancer cell proliferation and induce apoptosis in a subset of tumor cells with potency consistent with the intracellular inhibition of Akt kinase activity [45]. 5-*O*-(N-Boc-l-alanine)-renieramycin T and hydroquinone 5-O-cinnamoyl ester of renieramycin M, a series of renieramycin derivatives, showed CSC-suppression activities by inhibiting Akt signaling, resulting in the downregulation of CSC transcription factors (Nanog, Oct4, and Sox2) [18,32]. In this study, DH_25, a derivative of the RT right-half analog, efficiently inhibited and induced the death of lung CSCs (Figure 4A). Furthermore, the results of Western blot analysis declared a decrease in CSC marker CD133, CSC transcription factor Nanog, and CSC-related oncoprotein c-Myc (Figure 4B). Remarkably, DH_25 also downregulated the crucial upstream signaling of the other mentioned proteins, Akt and p-Akt protein levels (Figure 4B). Moreover, the decrease in the p-Akt/Akt ratio suggested that DH_25 might inactivate the Akt protein. As Akt is the main upstream regulator of cancer survival, Akt inhibition may lead to CSC suppression and apoptosis.

Several Akt inhibitors targeting protein binding sites have been developed and are currently being evaluated in clinical trials [46]. ATP-competitive inhibitors are among the most commonly used Akt inhibitors and significantly suppress Akt activity. These inhibitors bind directly to active Akt’s ATP-binding pocket in a PH-out conformation, resulting in paradoxical increases in Akt phosphorylation at threonine and serine residues, which have been reported to have noncatalytic functions [47]. Since the ATP-binding site is highly conserved in many protein kinases, these drugs are not very selective [48]. Allosteric inhibitors have been developed to improve Akt selectivity. They form intramolecular interactions with Akt residues in the linker region to stabilize Akt in the PH-in conformation and prevent Akt phosphorylation [49]. Several studies have revealed that allosteric inhibitors have higher specificity and fewer side effects, and some of them, such as MK2206, have been studied in clinical trials [50]. In this study, considering previous results that DH_25 could downregulate Akt, molecular docking simulations were performed using the binding interaction pattern of DH_25 with the ATP-binding site, allosteric binding site, and PH domain of Akt. The molecular docking result revealed that DH_25 bound within the allosteric binding site and formed a series of VDW energies suitable for potential interaction with Akt. DH_25 forms VDW interactions with Asn53, Gln59, Leu78, Gln79, Leu202, Lys268, and Trp80, with Trp80 exhibiting the most favorable interaction (−9.287 kcal/mol) (Figure 6). This may suggest that targeting Trp80 residues is the most critical factor in determining activity. By contrast, numerous allosteric Akt inhibitors exhibit more favorable interactions with specific Trp80 residues [18,51]. In addition, DH_25 showed a similar binding pattern to miransertib, an oral allosteric Akt-1 inhibitor, by VDW interaction with Trp80. Consequently, the findings imply that DH_25 could interact with Akt-1 through an allosteric mechanism.

Since there are other CSC regulatory pathways, including Wnt, Notch, and Hedgehog [52,53,54], the molecular docking was performed to investigate whether DH_25 could induce CSC death only through the Akt signaling pathway, or not. The γ-secretase, a Notch pathway component [55,56], and SMO, a regulatory protein involved in the Wnt and Hedgehog pathways [57,58,59,60,61,62], were used as DH_25 binding targets, while their inhibitors were used as references. Based on the observed interaction patterns, the results suggested that when compared to the references, DH_25 may not bind effectively to the γ-secretase and SMO binding sites and may have a limited or no effect on these pathways (Figures S1 and S2). Thus, these findings suggested that the CSC inhibition effects of DH_25 depended on its Akt targeting activity.

As a results, the downstream of Akt signaling, including the CSC markers Nanog and CD133, decreased (Figure 4B). CD133 and Nanog are two important markers of CSCs that are regulated by the Akt signaling pathway. Activation of the Akt signaling pathway can lead to the phosphorylation and activation of transcription factors such as Nanog, which can then bind to the CD133 promoter and activate its expression [63]. In addition, the Akt signaling pathway can promote the stabilization of CD133 protein by inhibiting its degradation, which can contribute to the maintenance of CSC properties [64]. To support this concept, LY294002, a PI3K/Akt pathway inhibitor, was used in the combination treatment. Having shown that Akt is a potential protein target of DH_25, it was found that the addition of the known PI3K/Akt inhibitor LY294002 enhanced the downregulation of Nanog and CD133 caused by DH_25 (Figure 8). Importantly, the upstream p-Akt was found to be completely diminished in response to the combination treatment. Therefore, it could be suggested that the CSC markers, Nanog and CD133, were regulated by the Akt signal.

By investigating and the molecular mechanism and anticancer effects of DH_25, insights into the potential therapeutic applications of the studied compound for cancer treatment can be gained. However, it is important to note that the results of in vitro experiments need to be validated with in vivo experiments to confirm their clinical relevance. Moreover, these findings may be useful in emphasizing DH_25 as a lead compound with supportive data that needs to be developed further for targeted anti-cancer approaches, and information on the SARs of right half analogs of RT may be useful in the development of RT-related compounds.

## 4. Materials and Methods

### 4.1. Reagents and Antibodies

Roswell Park Memorial Institute (RPMI) 1640 medium, fetal bovine serum (FBS), l-glutamine, antibiotic-antimycotic, trypsin-EDTA, and phosphate-bueredsaline (PBS) for cell culture were obtained from Gibco (Grand Island, NY, USA). Hoechst 33342, propidium iodide (PI), dimethyl sulfoxide (DMSO), 3-(4,5-dimethylthiazol-2-yl)-2,5-diphenyltetrazoliumbromide (MTT), crystal violet, LY294002, and bovine serum albumin (BSA) were obtained from Sigma-Aldrich, Co. (St. Louis, MO, USA). RIPA lysis buffer, primary antibodies (Mcl-1 (#94296), PARP (#9532), Bax (#5023), Bcl-2 (#4223), Akt (#9272), p-Akt (Ser473, #4060), c-Myc (#18583), Nanog (#4903), and β-actin (#4970), and secondary antibodies (anti-rabbit IgG (#7074) were obtained from Cell Signaling Technology (Danvers, MA, USA). CD133 primary antibody (ab19898) was acquired from Abcam (Waltham, MA, USA). Annexin V-FITC/PI apoptosis kit was purchased from ImmunoTools (Gladiolenweg 2, Friesoythe, Germany).

### 4.2. Cell Lines and Culture

Human non-small cell lung cancer (NSCLC) cell lines (H460 and H23) were obtained from the American Type Culture Collection (ATCC; Manassas, VA, USA) and were cultured in Roswell Park Memorial Institute (RPMI) 1640 medium, which was supplemented with 10% FBS, 2 mM L-glutamine, and 100 units/mL of antibiotic-antimycotic. The cells were maintained at 37 °C in a humidified incubator of 5% carbon dioxide (CO_2_). 85% confluence of cells was utilized for experiments in this study.

### 4.3. Derivatives of RT Right-Half Analog Synthesis

Derivatives of RT right-half analogs in this study, which are DH_16, DH_19, DH_22, DH_24, and DH_25, were newly synthesized from L-Tyr [19] as described in Supplementary Materials, including Supplemental Information 2 and Figures S3–S5. TM-(–)-4a was synthesized according to the previous research [19]. All compounds were confirmed to have 99% enantiomeric excess by HPLC analysis to prove that there is no racemization at the chiral center in the synthetic route. The ^1^H-NMR and ^13^C-NMR values were presented in Figures S4 and S5.

### 4.4. Preparation of Compound Stock Solutions

Derivatives of RT right-half analogs (DH_16, DH_19, DH_22, DH_24, DH_25, and TM-(–)-4a) were prepared as a 50 mM master stock solution by dissolving them in DMSO. The stock solutions were stored at −20 °C and freshly diluted with RPMI, to create a complete medium with required concentrations before treatment. The final concentrations of DMSO in all treatment conditions were 0.2% *v/v*, which showed no sign of cytotoxicity.

### 4.5. Cell Viability Assay

The MTT assay was used to evaluate the cell viability by measuring cellular metabolic activity, which is a sign of live cells. For the MTT assay, 1 × 10^4^ cells per well of NSCLC cells (H460 and H23) were seeded into 96-well plates and incubated overnight. After that, cells were treated with several concentrations (0–100 µM) of derivatives of the RT right-half analog (DH_16, DH_19, DH_22, DH_24, DH_25, and TM-(–)-4a) for 24 h at 37 °C and analyzed by the MTT assay according to the manufacturer’s protocol (Sigma Chemical, St. Louis, MO, USA). After removing the medium containing treatment compounds, 0.5 mg/mL of MTT solution was added and then incubated for 3 h at 37 °C in the dark. Next, MTT was removed, and 100% of the DMSO was replaced to dissolve the formazan crystals. A microplate reader (Anthros, Durham, NC, USA) was used to measure the optical density at 570 nm. The percentage of cell viability and the half maximal inhibitory concentration (IC_50_) were calculated according to the manufacturer’s protocol (7sea Biotech).

### 4.6. Nuclear Staining Assay

A nuclear staining assay was applied to define apoptotic and necrotic cell death by using nuclear staining with Hoechst 33342 and propidium iodide (PI) double staining. NSCLC cells (H460 and H23) were seeded into 96-well plates at a density of 1 × 10^4^ cells per well and incubated overnight. The cells were treated with several concentrations (0–50 µM) of derivatives of the RT right-half analog (DH_16, DH_19, DH_22, DH_24, and DH_25) for 24 h at 37 °C. Afterward, the cells were stained with 10 µg/mL of Hoechst 33342 and 5 µg/mL of PI for 15 min at 37 °C. Then, the fluorescence of cells was visualized and imaged under a fluorescence microscope (Nikon ECLIPSE Ts2, Tokyo, Japan). The percentage of apoptotic cells was analyzed and reported.

### 4.7. Colony Formation Assay

A colony formation assay was performed to investigate the ability of a single cancer cell to survive and grow into a colony in response to compound treatment. For overnight culture, a single-cell suspension of NSCLC cells (H460 and H23) was placed into 6-well plates at a density of 300 cells/well. Then, the cells were treated with several concentrations of DH_25 (0–10 µM) for 24 h. After that, the medium was replaced, and the cells were further cultured for 7 days at 37 °C with 5% CO_2_. 4% paraformaldehyde and 0.05% *w*/*v* of crystal violet were used to fix and stain the colonies for calculating the colony number.

### 4.8. Annexin V-FITC/PI Staining Apoptotic Assay

Annexin V-FITC/PI apoptosis kit (ImmunoTools, Gladiolenweg 2, Friesoythe, Germany) was introduced to investigate apoptotic cell death by using flow cytometry with annexin V-FITC/PI staining. NSCLC cells (H460 and H23) were seeded into 24-well plates at a density of 5 × 10^4^ cells/well and incubated overnight. The cells were treated with several concentrations of DH_25 (0–50 µM) and incubated for 24 h at 37 °C. After that, the cells were detached from the surface by trypsin-EDTA (0.25%). The cells were collected and incubated with 5 µL of annexin V-FITC and 1 µL of PI for 15 min at room temperature in the dark. Then, the cells were analyzed using GuavaCyte flow cytometry systems (GuavaSoft Software version 3.3, Merck, Burlington, MA, USA).

### 4.9. Western Blot Analysis

Protein expression levels in the cells were determined by Western blot analysis. NSCLC cells (H460 and H23) were seeded into 6-well plates at a density of 4 × 10^5^ cells/well for overnight treatment and incubated for 24 h at 37 °C with several concentrations of DH_25 (0–10 µM). In the conditions that LY294002 (5 µM) was used, it was added to pretreat the cells for 0.5 h before treatment with DH_25. After treatment, the floating cells known as apoptosis cells were collected by centrifuging at 1500 rpm for 5 min and aspirating the supernatants. The cells were lysed with RIPA lysis buffer containing 25 mM Tris-HCl, pH 7.6, 1% Nonidet P-40, 150 mM NaCl, 0.1% SDS, and 1% sodium deoxycholate for 30 min at 4 °C. The lysates were collected, and protein concentrations were determined by the BCA protein assay kit (Pierce Biotechnology, Rockford, IL, USA). SDS-PAGE was used to separate an equal amount of proteins from each sample, which were then transferred to 0.2-µm polyvinylidene difluoride membranes. Then, the blots were blocked with 5% skim milk in Tris-buffered saline/Tween 20 (containing 25 mM Tris-HCl, pH 7.5, 125 mM NaCl, and 0.1% Tween 20) for 2 h and incubated with primary antibodies specific to PARP, Mcl-1, Bcl-2, Bax, CD133, Akt, c-Myc, Nanog, and β-actin, overnight at 4 °C. After that, the membranes were washed with Tris-buffered saline/Tween 20 three times and incubated with secondary antibodies for 2 h at room temperature. The protein bands were detected with the enhanced chemiluminescent detection system (SupersignalWest Pico, Pierce, Rockford, IL, USA) and exposed to X-ray film. The intensity of protein bands was analyzed by ImageJ software (version 1.52, National Institutes of Health, Bethesda, MD, USA). Densitometric values were calculated relative to β-actin.

### 4.10. Three-Dimensional (3D) Spheroid Formation Assay

A three-dimensional (3D) spheroid formation assay was used to investigate the CSC subpopulation in cancer cells. NSCLC cells (H460 and H23) were seeded into 6-well ultralow-attachment plates at a density of 3 × 10^3^ cells/well and allowed to form a primary spheroid for 7 days. After incubation time, primary spheroids were resuspended into single cells and seeded into 24-well ultralow-attachment plates for 14 days to form secondary spheroids. Then, secondary spheroids were collected, and one single spheroid was transferred to 96-well ultralow-attachment plates. The spheroids were treated with DH_25 (0–10 µM) at 37 °C for 3 days. During the incubation time, the spheroids were visualized and imaged under the fluorescence microscope (Nikon ECLIPSE Ts2, Tokyo, Japan) every day. After 3 days, the spheroids were co-stained with Hoechst 33342 and PI at 37 °C for 15 min and then imaged using the fluorescence microscope (Nikon ECLIPSE Ts2, Tokyo, Japan).

### 4.11. Molecular Docking Analysis

The receptor structures of Akt1 with an ATP-competitive inhibitor (PDB ID 4GV1) [65], Akt1 with an allosteric inhibitor (PDB ID 5KCV) [66], the PH domain of Akt-1 (PDB ID 1UNQ) [67], γ-secretase (PDB ID 6LR4) [55], and SMO (PDB ID 5L7I) [68] were obtained from the Research Collaboratory for Structural Bioinformatics Protein Data Bank [69]. The missing residues in the protein structures were modeled using the Modeller 10.3 program [70] with the Discrete Optimized Protein Energy (DOPE) method. The 3D structure of DH_25 was built using MarvinSketch and optimized using the B3LYP/6-31G (d,p) basis set implemented in the Gaussian 09 program [71]. The UCSF Chimera 11.4 [72] was used to add hydrogen atoms to the protein, assign the Amber ff99SB force field to the receptor, and charge the ligand with AM1-BCC. DOCK 6.9 was used to dock DH_25 to the binding site using the standard flexible docking protocol [73]. For comparison, the grid energy scores (the van der Waals (VDW) energy + electrostatic (ES) energy) were also performed on the docked poses. The lower value of the grid score shows better interaction with the receptor. Additionally, 3D binding interactions were shown using the UCSF ChimeraX [74].

### 4.12. Statistical Analysis

The mean ± standard deviation (SD) of all data from at least three independently replicated experiments (*n* = 3) was established. An analysis of variance (ANOVA) was used to compare statistical differences among several groups with a significance level of *p* < 0.05. For data analysis and graph generation, SPSS software version 16 (SPSS Inc., Chicago, IL, USA) and GraphPad prism (GraphPad Software, San Diego, CA, USA) were used.

## 5. Conclusions

In conclusion, this study presents a novel derivative of the RT right-half analog, DH_25, that exhibits potent anticancer activity comparable to that of its parent compound but with a smaller structure that makes it easier to synthesize. According to the SARs study, DH_25 inhibits Mcl-1 similarly to other derivatives with cyanide and benzene ring from the previous study [21]. The modification of the benzene ring in DH_25 into thiazole has no effect on the Mcl-1 suppressing activity. Moreover, DH_25 is found to induce CSC death by directly interacting with the allosteric binding site of Akt, the key upstream regulator of CSCs and cancer survival. Blocking of Akt leads to apoptosis induction and CSC inhibition due to the lowering of anti-apoptotic proteins Bcl-2, oncoprotein c-Myc, transcription factor Nanog, and CSC marker CD133. These findings may be useful in emphasizing DH_25 as a lead compound with supportive data to be developed further for targeted anti-cancer approaches, and information on the SARs of right-half analogs of RT could provide benefits regarding the development of RT-related compounds.

## Data Availability

The datasets used and/or analyzed during the current study are available from the corresponding author upon reasonable request.

## Supplementary Materials

The following supporting information can be downloaded at: https://www.mdpi.com/article/10.3390/ijms24065345/s1.
