# Supplementary material for: Novel Synthetic Derivative of Renieramycin T Right-Half Analog Induces Apoptosis and Inhibits Cancer Stem Cells via Targeting the Akt Signal in Lung Cancer Cells
Source: Int J Mol Sci. 2023 Mar 10;24(6):5345. doi: 10.3390/ijms24065345 (PMC10049402; doi:10.3390/ijms24065345)
Supplement: Supplementary file 1 [file ijms-24-05345-s001.zip › Supplemental information 1.pdf]

Supplemental information 1

Results

2.4 DH_25 Directly Interacts with Akt, the Upstream Regulatory Signal of CSC

CSC is a subpopulation of cancer cells thought to be responsible for tumor initiation, progression, and recurrence [1]. Therefore, understanding the signaling pathways involved in the regulation of CSC is important for the development of targeted therapies. Several signaling pathways, including Akt, Notch, Hedgehog, and Wnt/β-catenin, have been implicated in the regulation of CSC in NSCLC [2].

Even the docking results showed the effective binding between DH_25 and the allostreic site of Akt (Figure 6), which was according to the Western blotting results (Figure 4B), but there is still a question about whether DH_25 could interact with the key proteins in the other CSC regulatory pathways, including Wnt, Notch, and Hedgehog, or not. Therefore, the molecular docking was performed to investigate at this point.

The Notch signaling pathway plays a crucial role in maintaining the self-renewal capacity of CSC in NSCLC [3]. Inhibition of the Notch pathway has been shown to reduce CSC populations in NSCLC, making it a promising therapeutic target [3]. γ-secretase, an essential component of the Notch pathway, is also involved in the maintenance of CSC populations in NSCLC. Inhibition of γ-secretase has been shown to reduce CSC populations and sensitize NSCLC cells to chemotherapy and radiation therapy [4]. Semagacestat is a gamma-secretase inhibitor that has been investigated for its potential to inhibit the Notch signaling pathway [5]. Based on the molecular docking study, the co-crystal ligand semagacestat (PDB ID 6LR4) was found to reproduce the original docking conformation with an RMSD of 1.1617 Å, and the binding energies of DH_25 and the co-crystal ligand semagacestat have been reported in (Figures S1A and S1B). The study revealed that DH_25 was located at different parts of the γ-secretase binding site (Figure S1C). The DH_25 pose was placed between the side chains of Leu268 and Leu286, but it was unable to imitate the interaction patterns produced by the reference compound (Figure S1D). These findings suggest that DH_25 may not bind effectively to the γ-secretase binding site and could potentially have limited or no effect on the Notch signaling pathway.

The Hedgehog (Hh) and Wnt signaling pathways are important pathways that regulate various cellular processes, including cell growth, differentiation, and survival [6]. The Hh pathway is activated by binding of Hh ligands to their receptors, which leads to the activation of the transcription factor Gli and subsequent transcription of Hh target genes [7]. The Wnt pathway is activated by binding of Wnt ligands to their receptors, which leads to the stabilization of β-catenin and transcription of Wnt target genes [8]. SMO (smoothened) inhibitors are a class of drugs that specifically target the Hh pathway by inhibiting the activity of the SMO protein. Recent studies have revealed crosstalk between Hh and Wnt pathways [9]. SMO inhibitors have been found to downregulate the expression of β-catenin and its target genes in various cancer cell lines [10]. This suggests that SMO inhibitors may have a dual effect on cancer cells by simultaneously inhibiting both the Hh and Wnt pathways. Vismodegib is an FDA-approved small molecule inhibitor that targets the Hh signaling pathway by inhibiting the activity of SMO [11], and it has also been shown to affect the Wnt signaling pathway. Based on the molecular docking study, the co-crystal ligand vismodegib was found to reproduce the original docking conformation with an RMSD of 1.3545 Å, and the binding energies of DH_25 and the co-crystal ligand vismodegib have been reported in Figures S2A and S2B. The study found that DH_25 was located in a distinct region of the SMO binding site compared to vismodegib (Figure S2C). Specifically, the DH_25 pose was found to interact with Lys204, Ser205, Trp206, Trp207, Glu208, and Lys395, but it was unable to imitate the interaction patterns produced by vismodegib (Figure S2D). Based on the observed interaction patterns, DH_25 may have limited or no activity as a dual inhibitor of the Hh and Wnt pathways.

Molecular docking of γ-secretase-DH_25 interaction

DH_25 VS semagacestat at γ-secretase

A

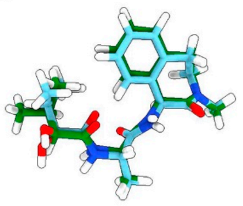

B

Compounds	Grid score (kcal/mol)	VDW energy (kcal/mol)	ES energy (kcal/mol)
Semagacestat (reference compound)	−52.514	−43.828	−8.686
DH_25	−43.885	−43.885	-

C

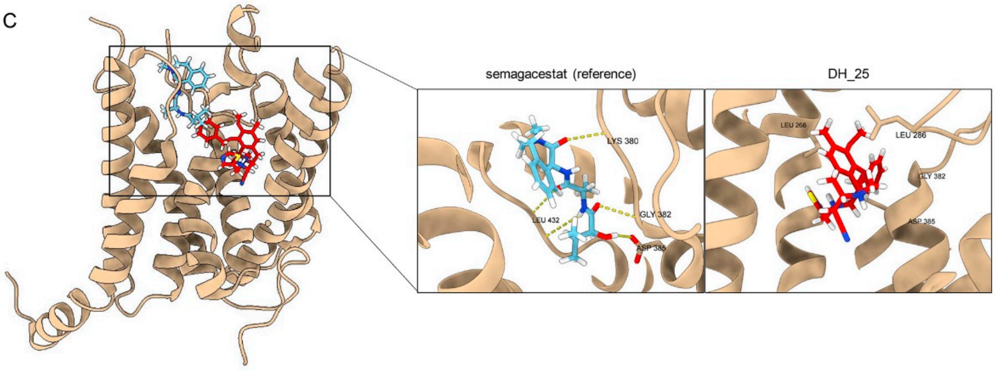

D

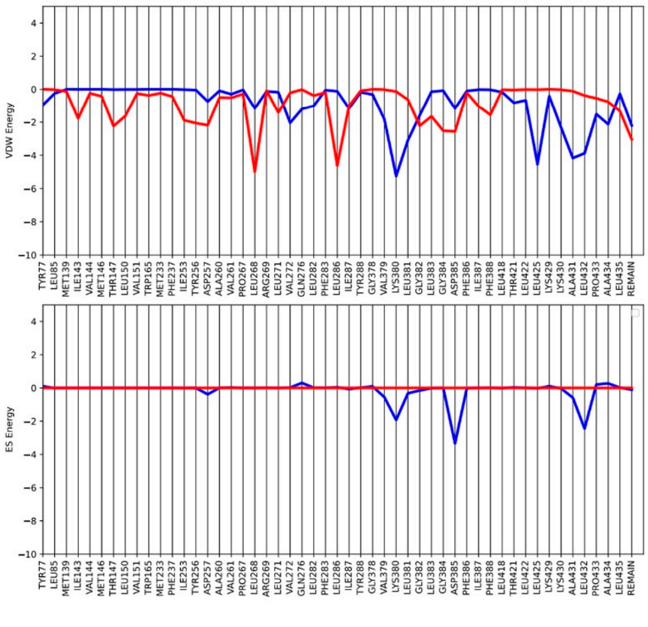

Figure S1. Molecular docking of DH_25 and γ-secretase compared with the γ-secretase inhibitor (semagacestat). **(A)** Structural superimposition of redocked (blue) and experimental native ligand (green) at the γ-secretase. **(B)** Binding

energies of the ligand in complex with the γ-secretase. **(C)** The γ-secretase in complexed with DH_25 or semagacestat reference. The yellow dashed lines denote hydrogen-bonding interaction. (D) Footprint analysis for DH_25 (red lines) compared to the semagacestat reference (blue lines) into the γ-secretase.

Molecular docking of SMO-DH_25 interaction

DH_25 VS vismodegib at SMO

A

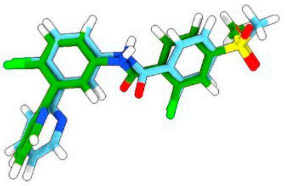

B

Compounds	Grid score (kcal/mol)	VDW energy (kcal/mol)	ES energy (kcal/mol)
Vismodegib (reference compound)	−55.932	−54.887	−1.045
DH_25	−27.264	−27.264	-

C

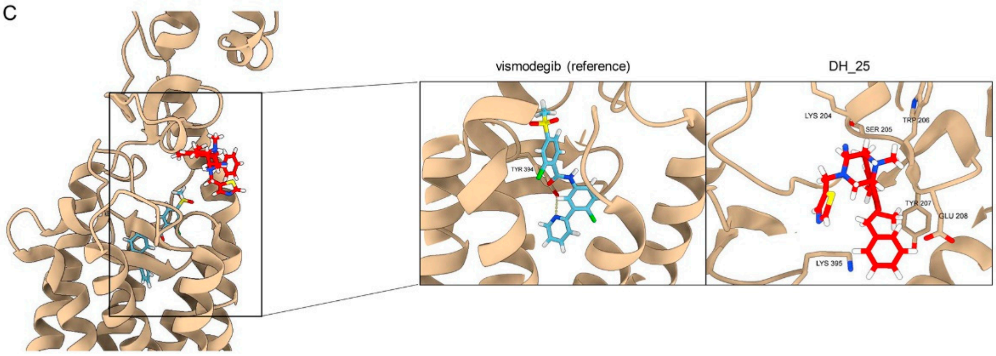

D

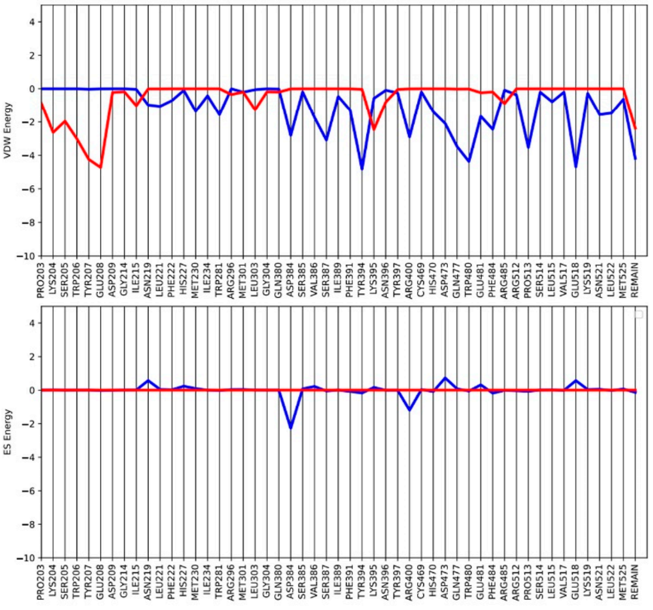

Figure S2. Molecular docking of DH_25 and SMO compared with SMO inhibitor (vismodegib). **(A)** Structural superimposition of redocked (blue) and experimental native ligand (green) at the SMO. **(B)** Binding energies of the ligand in complex with the SMO. **(C)** The SMO in complexed with DH_25 or vismodegib reference. The yellow dashed lines denote hydrogen-bonding interaction. (D) Footprint analysis for DH_25 (red lines) compared to the vismodegib reference (blue lines) into the SMO.

References

1. Ayob, A.Z.; Ramasamy, T.S. Cancer stem cells as key drivers of tumour progression. *Journal of biomedical science* **2018**, *25*, 20, doi:10.1186/s12929-018-0426-4.
2. Kumar, V.; Vashishta, M.; Kong, L.; Wu, X.; Lu, J.J.; Guha, C.; Dwarakanath, B.S. The Role of Notch, Hedgehog, and Wnt Signaling Pathways in the Resistance of Tumors to Anticancer Therapies. *Front Cell Dev Biol* **2021**, *9*, 650772, doi:10.3389/fcell.2021.650772.
3. Galluzzo, P.; Bocchetta, M. Notch signaling in lung cancer. *Expert Rev Anticancer Ther* **2011**, *11*, 533-540, doi:10.1586/era.10.158.
4. Capaccione, K.M.; Pine, S.R. The Notch signaling pathway as a mediator of tumor survival. *Carcinogenesis* **2013**, *34*, 1420-1430, doi:10.1093/carcin/bgt127.
5. Henley, D.B.; Sundell, K.L.; Sethuraman, G.; Dowsett, S.A.; May, P.C. Safety profile of semagacestat, a gamma-secretase inhibitor: IDENTITY trial findings. *Curr Med Res Opin* **2014**, *30*, 2021-2032, doi:10.1185/03007995.2014.939167.
6. Taipale, J.; Beachy, P.A. The Hedgehog and Wnt signalling pathways in cancer. *Nature* **2001**, *411*, 349-354, doi:10.1038/35077219.
7. Skoda, A.M.; Simovic, D.; Karin, V.; Kardum, V.; Vranic, S.; Serman, L. The role of the Hedgehog signaling pathway in cancer: A comprehensive review. *Bosn J Basic Med Sci* **2018**, *18*, 8-20, doi:10.17305/bjbms.2018.2756.
8. Liu, J.; Xiao, Q.; Xiao, J.; Niu, C.; Li, Y.; Zhang, X.; Zhou, Z.; Shu, G.; Yin, G. Wnt/β-catenin signalling: function, biological mechanisms, and therapeutic opportunities. *Signal Transduction and Targeted Therapy* **2022**, *7*, 3, doi:10.1038/s41392-021-00762-6.
9. Pietrobono, S.; Stecca, B. Targeting the Oncoprotein Smoothened by Small Molecules: Focus on Novel Acylguanidine Derivatives as Potent Smoothened Inhibitors. *Cells* **2018**, *7*, doi:10.3390/cells7120272.
10. Krishnamurthy, N.; Kurzrock, R. Targeting the Wnt/beta-catenin pathway in cancer: Update on effectors and inhibitors. *Cancer treatment reviews* **2018**, *62*, 50-60, doi:10.1016/j.ctrv.2017.11.002.
11. Rudin, C.M. Vismodegib. *Clinical cancer research : an official journal of the American Association for Cancer Research* **2012**, *18*, 3218-3222, doi:10.1158/1078-0432.Ccr-12-0568.
