# Supplementary material for: Novel Synthetic Derivative of Renieramycin T Right-Half Analog Induces Apoptosis and Inhibits Cancer Stem Cells via Targeting the Akt Signal in Lung Cancer Cells
Source: Int J Mol Sci. 2023 Mar 10;24(6):5345. doi: 10.3390/ijms24065345 (PMC10049402; doi:10.3390/ijms24065345)
Supplement: Supplementary file 1 [file ijms-24-05345-s001.zip › Supplemental information 2.pdf]

Supplemental information 2

Materials and Methods

4.3 Derivatives of RT Right-Half Analog Synthesis

The asymmetric synthesis of DH_16, DH_19, DH_22, DH_24, and DH_25 having pyridine, pyrimidine, or thiazole were performed in two steps from the common intermediate **1**, which can be easily synthesized from L-Tyr [1] (Figure S3). Therefore, after activation of the lactam nitrogen with sodium hydride, in situ prepared bromide was treated to obtain the corresponding *N*-alkylated compounds **2**. The lactam carbonyl of compound **2** was partially reduced with $LiAlH_2(OEt)_2$ in THF to generate the aminal, which was then treated with KCN and acetic acid to give the aminonitrile **3** as a single diastereomer. In the case of **2d** having a pyrimidine ring, the pyrimidine ring was also reduced under reducing conditions along with the lactam carbonyl to give aminonitrile **3d** (DH_24) with a dihydropyrimidine ring. The ^{1}H-NMR and ^{13}C-NMR values were presented in Figures S4 and S5.

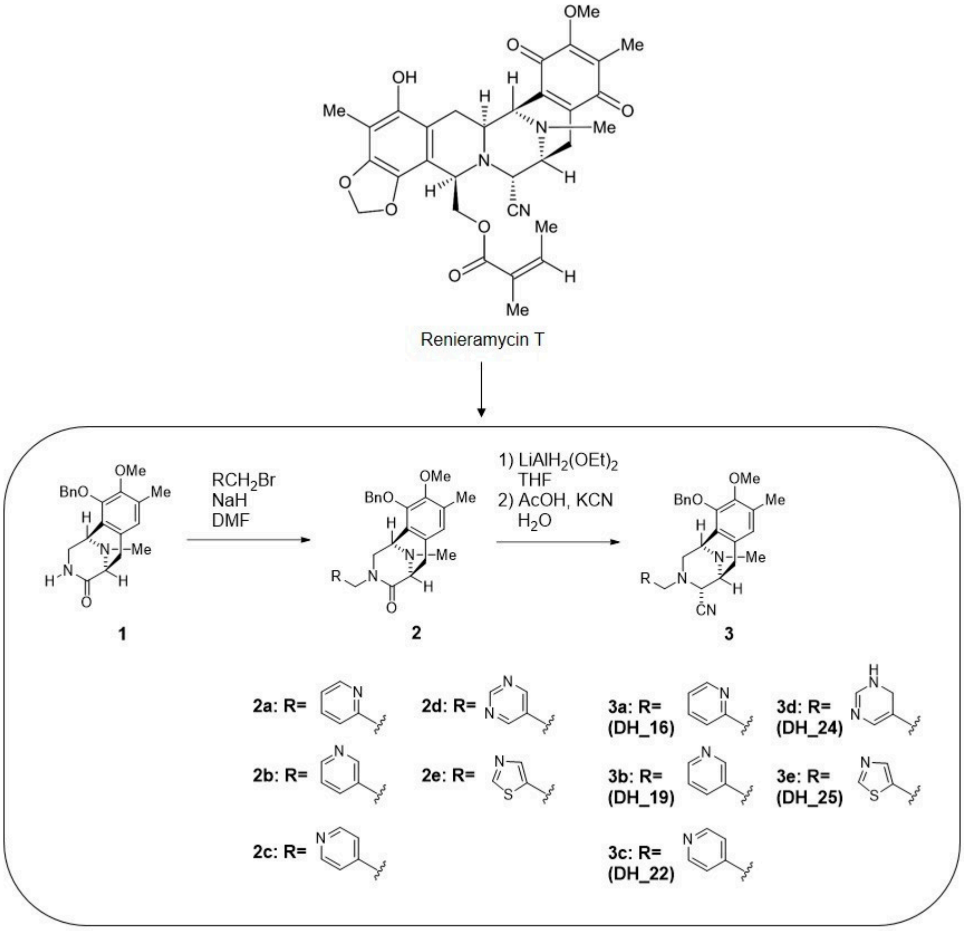

Figure S3. Asymmetric synthesis of derivatives of RT right-half analog (DH_16, DH_19, DH_22, DH_24, and DH_25)

*4.3.1. (1R,5S)-10-(benzyloxy)-9-methoxy-8,11-dimethyl-3-(pyridin-2-ylmethyl)-2,3,5,6-tetrahydro-1,5-epiminobenzo[d]azocin-4(1H)-one **(2a)***

To 2-pyridinemethanol (210 μL, 2.18 mmol) was added 49% hydrobromic acid (2.1 mL). The solution was refluxed for 3.5 h. The solution was concentrated, and the obtained residue was diluted with saturated $NaHCO_3$ solution (20 mL) and extracted with Et_2O (3×20 mL). The organic layer was washed with brine (20 mL), dried over Na_2SO_4, and filtered. The solvent was removed under reduced pressure and the crude product was dissolved in anhydrous dimethyl formamide (2.0 mL) for the next step. To a solution of

NaH (60% oil dispersion, 63.5 mg, 1.56 mmol) in DMF (1.0 mL) was slowly added lactam **2** (110 mg, 311 μmol) in DMF (3.0 mL) over 10 min at 0 °C. The reaction mixture was stirred for 30 min at 0 °C, after which the above bromide solution was added dropwise over 10 min. The reaction mixture was stirred for 14 h at 25 °C. The reaction mixture was diluted with H_2O (70 mL) and saturated potassium sodium tartrate solution (70 mL), and extracted with $CHCl_3$ (3×70 mL). The combined extracts were washed with brine (70 mL), dried over Na_2SO_4, and concentrated in vacuo to give a residue. The residue was purified by SiO_2 flash column chromatography ($CHCl_3$:MeOH = 19:1) to afford compound **2a** (111 mg, 80%) as a yellow gummy.

2a: $[\alpha]_D^{24}$ −90.0 (*c* 0.64, $CHCl_3$). ^{1}H-NMR (400 MHz, $CDCl_3$) δ: 8.44-8.42 (1H, m), 7.38-7.25 (6H, m), 7.06-7.03 (1H, m), 6.76 (1H, s), 6.45 (1H, d, *J* = 7.8 Hz), 5.00 (1H, d, *J* = 11.4 Hz), 4.93 (1H, d, *J* = 11.4 Hz), 4.87 (1H, d, *J* = 15.6 Hz), 4.34 (1H, d, *J* = 15.6 Hz), 3.92 (1H, d, *J* = 4.6 Hz), 3.85 (1H, dd, *J* = 11.7, 4.6 Hz), 3.73 (3H, s), 3.67 (1H, d, *J* = 6.4 Hz), 3.18 (1H, dd, *J* = 17.1, 6.4 Hz), 3.05 (1H, dd, *J* = 11.7, 0.9 Hz), 2.86 (1H, d, *J* = 17.1 Hz), 2.33 (3H, s), 2.29 (3H, s). ^{13}C-NMR (100 MHz, $CDCl_3$) δ: 170.4, 156.8, 149.5, 149.1, 148.4, 137.3, 136.5, 131.4, 128.6, 128.5, 128.1, 128.1, 126.3, 125.7, 122.0, 120.6, 74.3, 60.0, 59.4, 51.7, 51.6, 51.1, 39.8, 27.6, 15.7. IR (KBr) cm^{-1}: 2937, 1653, 1591, 1571, 1487, 1336, 1230, 1175, 1143, 1059, 1010, 902, 748, 700, 667, 620, 504, 418, 407. EI-MS *m/z* (%): 444 (M^++1, 11),443 (M^+, 38), 352 (15), 351 (19), 295 (23), 294 (100), 293 (10), 204 (40), 203 (36). HR-EI-MS *m/z*: 443.2209 (M^+, calcd for $C_{27}H_{29}N_3O_3$, 443.2211).

*4.3.2. (1R,5S)-10-(benzyloxy)-9-methoxy-8,11-dimethyl-3-(pyridin-3-ylmethyl)-2,3,5,6-tetrahydro-1,5-epiminobenzo[d]azocin-4(1H)-one (**2b**)*

To 3-pyridinemethanol (210 μL, 2.18 mmol) was added 49% hydrobromic acid (2.1 mL). The solution was refluxed for 4 h. The solution was concentrated, and the obtained residue was diluted with saturated $NaHCO_3$ solution (20 mL) and extracted with Et_2O (3×20 mL). The organic layer was washed with brine (20 mL), dried over Na_2SO_4, and filtered. The solvent was removed under reduced pressure and the crude product was dissolved in anhydrous dimethyl formamide (2.0 mL) for the next step. To a solution of NaH (60% oil dispersion, 63.5 mg, 1.56 mmol) in DMF (2.0 mL) was slowly added lactam **2** (110 mg, 313 μmol) in DMF (2.0 mL) over 10 min at 0 °C. The reaction mixture was stirred for 20 min at 0 °C, after which the above bromide solution was added dropwise over 10 min. The reaction mixture was stirred for 16 h at 25 °C. The reaction mixture was diluted with H_2O (60 mL) and saturated potassium sodium tartrate solution (40 mL), and extracted with $CHCl_3$ (3×60 mL). The combined extracts were washed with brine (60 mL), dried over Na_2SO_4, and concentrated in vacuo to give a residue. The residue was purified by SiO_2 flash column chromatography ($CHCl_3$:MeOH = 9:1) to afford compound **2b** (72.7 mg, 58%) as a yellow gummy.

2b: $[\alpha]_D^{24}$ −85.8 (*c* 0.32, $CHCl_3$). ^{1}H-NMR (400 MHz, $CDCl_3$) δ: 8.34 (1H, t, *J* = 3.1 Hz), 8.23 (1H, s), 7.37-7.25 (5H, m), 6.98-6.98 (2H, m), 6.74 (1H, s), 4.99 (1H, d, *J* = 11.6 Hz), 4.92 (1H, d, *J* = 11.6 Hz), 4.74 (1H, d, *J* = 15.1 Hz), 4.20 (1H, d, *J* = 15.1 Hz), 3.87 (1H, d, *J* = 4.4 Hz), 3.73-3.66 (2H, m), 3.71 (3H, s), 3.16 (1H, dd, *J* = 17.0, 6.3 Hz), 2.90 (1H, d, *J* = 11.7 Hz), 2.83 (1H, d, *J* = 17.0 Hz), 2.29 (3H, s), 2.27 (3H, s). ^{13}C-NMR (100 MHz, $CDCl_3$) δ: 170.4, 149.5, 149.1, 148.6, 148.3, 137.3, 134.8, 132.2, 131.6, 128.6, 128.3, 128.2, 125.7, 125.7, 123.4, 74.3, 60.1, 59.3, 51.6, 51.1, 46.5, 39.7, 27.2, 15.8. IR (KBr) cm^{-1}: 3008, 2939, 1644, 1487, 1446, 1336, 1216, 1059, 1010, 749, 700, 667, 431, 423, 409. EI-MS *m/z* (%): 443 (M^+, 24), 295 (22), 294 (100), 204 (30), 203 (34). HR-EI-MS *m/z*: 443.2209 (M^+, calcd for $C_{27}H_{29}N_3O_3$, 443.2206).

*4.3.3. (1R,5S)-10-(benzyloxy)-9-methoxy-8,11-dimethyl-3-(pyridin-4-ylmethyl)-2,3,5,6-tetrahydro-1,5-epiminobenzo[d]azocin-4(1H)-one (**2c**)*

To 3-pyridinemethanol (0.43 g, 3.94 mmol) was added 49% hydrobromic acid (4.3 mL). The solution was refluxed for 4 h. The solution was concentrated, and the obtained residue was diluted with saturated $NaHCO_3$ solution (40 mL) and extracted with Et_2O (3×40 mL). The organic layer was washed with brine (40 mL), dried over Na_2SO_4, and filtered. The solvent was removed under reduced pressure and the crude product was dissolved in anhydrous dimethyl formamide (4.0 mL) for the next step. To a solution of NaH (60% oil dispersion, 117 mg, 2.84 mmol) in DMF (4.0 mL) was slowly added lactam **2** (202 mg, 567 μmol) in DMF (4.0 mL) over 10 min at 0 °C. The reaction mixture was stirred for 30 min at 0 °C, after which the above bromide solution was added dropwise over 10 min. The reaction mixture was stirred for 12 h at 25 °C. The

reaction mixture was diluted with H_2O (30 mL), and extracted with $CHCl_3$ (3×60 mL). The combined extracts were washed with brine (60 mL), dried over Na_2SO_4, and concentrated in vacuo to give a residue. The residue was purified by SiO_2 flash column chromatography ($CHCl_3$:MeOH = 9:1) to afford compound **2c** (217 mg, quant.) as a yellow gummy.

2c: $[\alpha]_D^{24}$ −115.0 (*c* 0.14, $CHCl_3$). ^{1}H-NMR (400 MHz, $CDCl_3$) δ: 8.28-8.27 (2H, m), 7.36-7.25 (5H, m), 6.79 (1H, s), 6.56-6.55 (2H, m), 4.98 (2H, s), 4.97 (1H, d, *J* = 16.0 Hz), 3.96 (1H, d, *J* = 16.0 Hz), 3.88 (1H, d, *J* = 4.1 Hz), 3.76-3.68 (2H, m), 3.71 (3H, s), 3.18 (1H, dd, *J* = 17.1, 6.2 Hz), 2.92 (1H, dd, *J* = 11.6, 1.3 Hz), 2.84 (1H, d, *J* = 17.1 Hz), 2.32 (3H, s), 2.30 (3H, s). ^{13}C-NMR (100 MHz, $CDCl_3$) δ: 170.6, 149.7, 149.7, 148.4, 145.8, 137.3, 131.7, 128.5, 128.4, 128.3, 128.2, 125.9, 125.8, 121.8, 74.4, 60.1, 59.4, 51.7, 51.6, 47.8, 39.7, 27.2, 15.8. IR (KBr) cm^{-1}: 3008, 2940, 1647, 1604, 1488, 1446, 1415, 1336, 1216, 1059, 1009, 901, 748, 700, 667, 472, 419, 409, 404. EI-MS *m*/*z* (%): 443 (M^+, 26), 295 (22), 294 (100), 204 (32), 203 (36), 91 (10). HR-EI-MS *m*/*z*: 443.2209 (M^+, calcd for $C_{27}H_{29}N_3O_3$, 443.2212).

*4.3.4. (1R,5S)-10-(benzyloxy)-9-methoxy-8,11-dimethyl-3-(pyrimidin-5-ylmethyl)-2,3,5,6-tetrahydro-1,5-epiminobenzo[d]azocin-4(1H)-one (**2d**)*

To a solution of 5-(Hydroxymethyl)pyrimidine (264 mg, 2.33 mmol) in CH_2Cl_2 (mL) was added tetrabromomethane (849 mg, 2.56 mmol) and triphenylphosphine (671 mg, 2.56 mmol) at 0 °C. The reaction mixture was stirred for 1 h at 25 °C. The reaction mixture was diluted with H_2O (100 mL) and saturated potassium sodium tartrate solution (50 mL), and extracted with EtOAc (3×100 mL). The combined extracts were washed with brine (70 mL), dried over Na_2SO_4, and concentrated in vacuo to give a residue, and the crude product was dissolved in anhydrous dimethyl formamide (2.0 mL) for the next step. To a solution of NaH (60% oil dispersion, 66.4 mg, 1.59 mmol) in DMF (2.0 mL) was slowly added lactam **2** (109 mg, 308 μmol) in DMF (2.0 mL) over 10 min at 0 °C. The reaction mixture was stirred for 30 min at 0 °C, after which the above bromide solution was added dropwise over 10 min. The reaction mixture was stirred for 14 h at 25 °C. The reaction mixture was diluted with H_2O (60 mL) and extracted with $CHCl_3$ (3×60 mL). The combined extracts were washed with brine (60 mL), dried over Na_2SO_4, and concentrated in vacuo to give a residue. The residue was purified by SiO_2 flash column chromatography ($CHCl_3$:MeOH = 19:1) to afford compound **2d** (113 mg, 83%) as a pale yellow oil.

2d: $[\alpha]_D^{24}$ −96.8 (*c* 1.25, $CHCl_3$). ^{1}H-NMR (400 MHz, $CDCl_3$) δ: 9.01 (1H, s), 8.22 (2H, s), 7.38-7.28 (5H, m), 6.74 (1H, s), 5.09 (1H, d, *J* = 11.4 Hz), 4.99 (1H, d, *J* = 11.4 Hz), 4.67 (1H, d, *J* = 15.3 Hz), 4.21 (1H, d, *J* = 15.3 Hz), 3.85 (1H, d, *J* = 4.4 Hz), 3.76-3.72 (1H, m), 3.74 (3H, s), 3.66 (1H, d, *J* = 6.1 Hz), 3.15 (1H, dd, *J* = 17.2, 6.1 Hz), 2.95 (1H, d, *J* = 11.2 Hz), 2.78 (1H, d, *J* = 17.2 Hz), 2.29 (3H, s), 2.25 (3H, s). ^{13}C-NMR (100 MHz, $CDCl_3$) δ: 170.7, 157.7, 156.0, 149.6, 148.2, 137.3, 132.0, 130.2, 128.6, 128.4, 128.3, 128.1, 125.9, 125.4, 74.4, 60.1, 59.3, 51.7, 51.5, 44.8, 39.6, 26.8, 15.8. IR (KBr) cm^{-1}: 2938, 2463, 1726, 1653, 1562, 1410, 1336, 1173, 1059, 1009, 904, 744, 700, 664, 634, 472, 438, 418, 411. EI-MS *m*/*z* (%): 444 (M^+, 23), 295 (22), 294 (100), 204 (28), 203 (37), 91 (13). HR-EI-MS *m*/*z*: 444.2161 (M^+, calcd for $C_{26}H_{28}N_4O_3$, 444.2162).

*4.3.5. (1R,5S)-10-(benzyloxy)-9-methoxy-8,11-dimethyl-3-(thiazol-5-ylmethyl)-2,3,5,6-tetrahydro-1,5-epiminobenzo[d]azocin-4(1H)-one (**2e**)*

To a solution of 1,3-thiazol-5-ylmethanol (300 μL, 3.44 mmol) in $CHCl_3$ (5.6 mL) was added phosphorus tribromide (123 μL, 1.17 mmol) over 10 min at 25 °C. The reaction mixture was stirred for 1 h at 85 °C. The reaction mixture was diluted with 5% $NaHCO_3$ solution (50 mL) and extracted with CH_2Cl_2 (3×50 mL). The combined extracts were washed with H_2O (50 mL), dried over Na_2SO_4, and concentrated in vacuo to give a residue, and the crude product was dissolved in anhydrous dimethyl formamide (2.0 mL) for the next step.

To a solution of NaH (60% oil dispersion, 57.0 mg, 1.39 mmol) in DMF (2.0 mL) was slowly added lactam **2** (104 mg, 284 μmol) in DMF (2.0 mL) over 10 min at 0 °C. The reaction mixture was stirred for 30 min at 0 °C, after which the above bromide solution was added dropwise over 10 min. The reaction mixture was diluted with H_2O (50 mL) and saturated potassium sodium tartrate solution (40 mL), and extracted with $CHCl_3$ (3×50 mL). The combined extracts were washed with brine (50 mL), dried over Na_2SO_4, and concentrated in vacuo to give a residue. The residue was purified by SiO_2 flash column chromatography ($CHCl_3$:MeOH = 9:1) to afford compound **2e** (68.6 mg, 54%) as a yellow oil.

2e: $[\alpha]_D^{24}$ −81.1 (*c* 0.30, $CHCl_3$). ^{1}H-NMR (400 MHz, $CDCl_3$) δ: 8.59 (1H, s), 7.46 (1H, s), 7.39-7.30 (5H, m), 6.70 (1H, s), 5.03 (2H, d, *J* = 2.7 Hz), 4.71 (1H, d, *J* = 15.2 Hz), 4.50 (1H, d, *J* = 15.2 Hz), 3.88 (1H, d, *J* = 4,4 Hz), 3.76-3.72 (1H, m), 3.75 (3H, s), 3.61 (1H, d, *J* = 6.4 Hz), 3.12 (1H, dd, *J* = 17.1, 6.4 Hz), 3.01 (1H, dd, *J* = 11.4, 1.1 Hz), 2.78 (1H, d, *J* = 17.1 Hz), 2.26 (3H, s), 2.25 (3H, s). ^{13}C-NMR (100 MHz, $CDCl_3$) δ: 170.2, 153.6, 149.4, 148.3, 142.0, 137.4, 133.7, 131.6, 128.6, 128.2, 128.2, 125.8, 125.7, 74.3, 60.1, 59.2, 51.4, 50.9, 41.8, 39.7, 28.2, 15.8. IR (KBr) cm^{-1}: 3008, 2938, 2467, 1648, 1487, 1445, 1336, 1216, 1135, 1059, 1009, 874, 744, 701, 667, 602, 513, 413. EI-MS *m/z* (%): 449 (M^+, 26), 295 (22), 294 (100), 204 (32), 203 (35), 91 (11). HR-EI-MS *m/z*: 449.1773 (M^+, calcd for $C_{25}H_{28}N_3O_3S$, 449.1771).

*4.3.6. (1R,4R,5S)-10-(benzyloxy)-9-methoxy-8,11-dimethyl-3-(pyridin-2-ylmethyl)-1,2,3,4,5,6-hexahydro-1,5-epiminobenzo[d]azocine-4-carbonitrile [**3a** (**DH_16**)]*

To a solution of lactam **2a** (111 mg, 250 μmol) in THF (5.5 mL) at 0 °C was slowly added $LiAlH_2(OEt)_2$ (1.0 mol/L in CH_2Cl_2, 3. 0 mL, 3. 0 mmol, 12 eq.) over 10 min. The reaction mixture was stirred at 0 °C for 3 h. The reaction mixture was quenched with AcOH (300 μL, 5.25 mmol, 21 equiv.), followed by the addition of KCN (99.6 mg, 1.50 mmol, 6.0 equiv.) in H_2O (450 μL), and stirring was continued for 18 h at 25 °C. The reaction mixture was neutralized with 5% $NaHCO_3$ solution and diluted with saturated Rochell's salt aq., and the mixture was stirred for 1 h. The reaction mixture was extracted with $CHCl_3$ (3 × 100 mL). The combined extracts were washed with brine (100 mL), dried over Na_2SO_4, and concentrated in vacuo to give a residue. The residue was purified by SiO_2 flash column chromatography ($CHCl_3$:MeOH = 19:1) to afford compound **3a** (**DH_16**, 94.6 mg, 83%) as a colorless amorphous.

3a (**DH_16**): $[\alpha]_D^{24}$ −47.9 (*c* 1.63, $CHCl_3$). ^{1}H-NMR (400 MHz, $CDCl_3$) δ: 8.44 (1H, dt, *J* = 4.9, 0.9 Hz), 7.37-7.25 (6H, m), 7.04 (1H, dd, *J* = 7.3, 4.9 Hz), 6.72 (1H, s), 6.47 (1H, d, *J* = 8.0 Hz), 5.03 (1H, d, *J* = 11.4 Hz), 4.86 (1H, d, *J* = 11.4 Hz), 3.90 (1H, s), 3.82 (3H, s), 3.79 (1H, d, *J* = 1.8 Hz), 3.73 (1H, d, *J* = 15.2 Hz), 3.69 (1H, d, *J* = 15.2 Hz), 3.27 (1H, d, *J* = 7.6 Hz), 3.09 (1H, dd, *J* = 17.7, 7.6 Hz), 2.89 (1H, dd, *J* = 11.1, 2.9 Hz), 2.49-2.43 (2H, m), 2.32 (3H, s), 2.16 (3H, s). ^{13}C-NMR (100 MHz, $CDCl_3$) δ: 157.9, 149.1, 148.9, 148.3, 137.3, 136.3, 130.2, 130.1, 128.5, 128.5, 128.2, 126.6, 124.4, 122.0, 121.7, 116.6, 74.5, 60.9, 60.1, 60.1, 55.6, 53.2, 52.8, 41.3, 25.1, 15.8. IR (KBr) cm^{-1}: 3012, 2937, 2825, 1591, 1432, 1321, 1230, 1168, 1061, 1030, 900, 849, 804, 756, 700, 667, 481, 440, 424, 407. EI-MS *m/z* (%): 454 (M^+, 1), 295 (24), 294 (100), 204 (24), 203 (25). HR-EI-MS *m/z*: 454.2369 (M^+, calcd for $C_{28}H_{30}N_4O_2$, 454.2366).

*4.3.7. (1R,4R,5S)-10-(benzyloxy)-9-methoxy-8,11-dimethyl-3-(pyridin-3-ylmethyl)-1,2,3,4,5,6-hexahydro-1,5-epiminobenzo[d]azocine-4-carbonitrile [**3b** (**DH_19**)]*

To a solution of lactam **2b** (72.7 mg, 164 μmol) in THF (3.6 mL) at 0 °C was slowly added $LiAlH_2(OEt)_2$ (1.0 mol/L in CH_2Cl_2, 1.96 mL, 1.96 mmol, 12 equiv.) over 10 min. The reaction mixture was stirred at 0 °C for 3 h. The reaction mixture was quenched with AcOH (200 μL, 3.44 mmol, 21 equiv.), followed by the addition of KCN (64.0 mg, 983 μmol, 6.0 equiv.) in H_2O (300 μL), and stirring was continued for 16 h at 25 °C. The reaction mixture was neutralized with 5% $NaHCO_3$ solution and diluted with saturated Rochell's salt aq., and the mixture was stirred for 1 h. The reaction mixture was extracted with $CHCl_3$ (3 × 100 mL). The combined extracts were washed with brine (100 mL), dried over Na_2SO_4, and concentrated in vacuo to give a residue. The residue was purified by SiO_2 flash column chromatography ($CHCl_3$:MeOH = 19:1) to afford compound **3b** (**DH_19**, 56.6 mg, 76%) as a colorless amorphous.

3b (**DH_19**): $[\alpha]_D^{24}$ −38.6 (*c* 0.31, $CHCl_3$). ^{1}H-NMR (400 MHz, $CDCl_3$) δ: 8.43 (1H, dd, *J* = 4.7, 1.7 Hz), 8.20 (1H, d, *J* = 1.7 Hz), 7.37-7.27 (5H, m), 7.13 (1H, dt, *J* = 8.2, 1.7 Hz), 7.07 (1H, ddd, *J* = 8.2, 4.7, 0.8 Hz), 6.69 (1H, s), 5.04 (1H, d, *J* = 11.4 Hz), 4.92 (1H, d, *J* = 11.4 Hz), 3.89 (1H, s), 3.83 (3H, s), 3.66 (1H, d, *J* = 1.5 Hz), 3.55 (2H, s), 3.24 (1H, dd, *J* = 7.4, 1.5 Hz), 3.05 (1H, dd, *J* = 17.9, 7.4 Hz), 2.83 (1H, dd, *J* = 11.0, 2.9 Hz), 2.47 (1H, ddd, *J* = 11.0, 2.3, 1.1 Hz), 2.35 (1H, d, *J* = 17.9 Hz), 2.32 (3H, s), 2.14 (3H, s). ^{13}C-NMR (100 MHz, $CDCl_3$) δ: 149.7, 148.9, 148.8, 148.2, 137.4, 135.8, 132.6, 130.3, 129.9, 128.5, 128.5, 128.2, 126.3, 124.3, 123.3, 116.2, 74.5, 60.1, 59.3, 56.5, 55.3, 53.5, 52.7, 41.2, 25.1, 15.8. IR (KBr) cm^{-1}: 3019, 2937, 2829, 2403, 1483, 1427, 1322, 1217, 1162, 1062, 1027, 746, 668, 444, 434, 403. EI-MS *m/z* (%): 454 (M^+, 2), 295 (26), 294 (100), 243 (12), 204 (23), 203 (22). HR-EI-MS *m/z*: 454.2369 (M^+, calcd for $C_{28}H_{30}N_4O_2$, 454.2371).

*4.3.8. (1R,4R,5S)-10-(benzyloxy)-9-methoxy-8,11-dimethyl-3-(pyridin-4-ylmethyl)-1,2,3,4,5,6-hexahydro-1,5-epiminobenzo[d]azocine-4-carbonitrile [**3c** (**DH_22**)]*

To a solution of lactam **2c** (116 mg, 260 μmol) in THF (5.6 mL) at 0 °C was slowly added $LiAlH_2(OEt)_2$ (1.0 mol/L in CH_2Cl_2, 3.12 mL, 3.12 mmol, 12 equiv.) over 10 min. The reaction mixture was stirred at 0 °C for 3 h. The reaction mixture was quenched with AcOH (313 μL, 5.47 mmol, 21 equiv.), followed by the addition of KCN (102 mg, 1.56 mmol, 6.0 equiv.) in H_2O (600 μL), and stirring was continued for 19 h at 25 °C. The reaction mixture was neutralized with 5% $NaHCO_3$ solution and diluted with saturated Rochell's salt aq., and the mixture was stirred for 1 h. The reaction mixture was extracted with $CHCl_3$ (3 × 100 mL). The combined extracts were washed with brine (100 mL), dried over Na_2SO_4, and concentrated in vacuo to give a residue. The residue was purified by SiO_2 flash column chromatography ($CHCl_3$:MeOH = 19:1) to afford compound **3c** (**DH_22**, 106 mg, 89%) as a colorless amorphous.

3c (**DH_22**): $[\alpha]_D^{24}$ −49.9 (*c* 0.57, $CHCl_3$). ^{1}H-NMR (400 MHz, $CDCl_3$) δ: 8.35 (2H, d, *J* = 5.7 Hz), 7.33-7.25 (5H, m), 6.74-6.73 (3H, m), 5.03 (1H, d, *J* = 11.3 Hz), 4.94 (1H, d, *J* = 11.3 Hz), 3.87 (1H, s), 3.83 (3H, s), 3.70 (1H, s), 3.57 (1H, d, *J* = 15.0 Hz), 3.51 (1H, d, *J* = 15.0 Hz), 3.26 (1H, d, *J* = 7.6 Hz), 3.09 (1H, dd, *J* = 17.7, 7.6 Hz), 2.81 (1H, dd, *J* = 11.1, 2.9 Hz), 2.44-2.42 (1H, m), 2.41 (1H, d, *J* = 17.7 Hz), 2.34 (3H, s), 2.14 (3H, s). ^{13}C-NMR (100 MHz, $CDCl_3$) δ: 149.7, 149.0, 148.2, 146.6, 137.3, 130.3, 130.0, 128.5, 128.1, 126.3, 124.3, 122.9, 116.3, 74.5, 60.1, 59.8, 57.9, 55.4, 53.1, 52.7, 41.2, 25.1, 15.8. IR (KBr) cm^{-1}: 3012, 2938, 2829, 1602, 1483, 1444, 1415, 1366, 1322, 1217, 1165, 1062, 1029, 900, 810, 750, 700, 667, 490. EI-MS *m/z* (%): 454 (M^+, 2), 295 (26), 294 (100), 243 (12), 204 (22), 203 (20). HR-EI-MS *m/z*: 454.2369 (M^+, calcd for $C_{28}H_{30}N_4O_2$, 454.2371).

*4.3.9. (1R,4R,5S)-10-(benzyloxy)-3-({1,6-dihydropyrimidin-5-yl}methyl)-9-methoxy-8,11-dimethyl-1,2,3,4,5,6-hexahydro-1,5-epiminobenzo[d]azocine-4-carbonitrile [**3d** (**DH_24**)]*

To a solution of lactam **2d** (24.1 mg, 54.2 μmoll) in THF (1.2 mL) at 0 °C was slowly added $LiAlH_2(OEt)_2$ (1.0 mol/L in CH_2Cl_2, 651 μL, 651 μmol, 12 equiv.) over 10 min. The reaction mixture was stirred at 0 °C for 3 h. The reaction mixture was quenched with AcOH (65 μL, 1.14 mmol, 21 equiv.), followed by the addition of KCN (24.7 mg, 325 μmol, 6.0 equiv.) in H_2O (125 μL), and stirring was continued for 16 h at 25 °C. The reaction mixture was neutralized with 5% $NaHCO_3$ solution and diluted with saturated Rochell's salt aq., and the mixture was stirred for 1 h. The reaction mixture was extracted with $CHCl_3$ (3 × 30 mL). The combined extracts were washed with brine (30 mL), dried over Na_2SO_4, and concentrated in vacuo to give a residue. The residue was purified by SiO_2 flash column chromatography ($CHCl_3$:MeOH:Et_3N = 98:1:1) to afford compound **3d** (**DH_24**, 11.1 mg, 45%) as a colorless amorphous.

3d (**DH_24**): $[\alpha]_D^{24}$ −36.2 (*c* 0.42, $CHCl_3$). ^{1}H-NMR (500 MHz, $CDCl_3$) δ: 7.39-7.27 (5H, m), 6.93 (1H, s), 6.61 (1H, s), 5.94 (1H, s), 5.09 (1H, d, *J* = 11.3 Hz), 5.06 (1H, d, *J* = 11.3 Hz), 3.90 (1H, s), 3.83 (3H, s), 3.72 (1H, s), 3.59 (1H, d, *J* = 16.3 Hz), 3.32 (1H, d, *J* = 16.3 Hz), 3.24 (1H, d, *J* = 7.9 Hz), 3.02 (1H, dd, *J* = 17.8, 7.9 Hz), 2.80 (1H, d, *J* = 13.2 Hz), 2.75 (1H, d, *J* = 13.2 Hz), 2.70 (1H, dd, *J* = 11.1, 2.4 Hz), 2.46 (1H, d, *J* = 11.1 Hz), 2.35 (1H, d, *J* = 17.8 Hz), 2.26 (3H, s), 2.13 (3H, s). ^{13}C-NMR (125 MHz, $CDCl_3$) δ: 148.9, 148.2, 145.7, 137.5, 130.1, 130.0, 128.6, 128.5, 128.2, 126.3, 125.4, 124.3, 116.4, 108.1, 74.6, 60.2, 58.8, 57.4, 55.3, 53.4, 52.7, 44.3, 41.3, 25.1, 15.8. IR ($CHCl_3$) cm^{-1}: 3020, 2941, 2395, 1658, 1583, 1483, 1322, 1215, 1064, 1028, 748, 669, 587, 485, 464, 452, 441, 431, 425, 417, 411. HR-ESI-MS *m/z*: 458.2556 ($[M+H]^+$, calcd for $C_{27}H_{32}N_5O_2$, 458.2549).

*4.3.10. (1R,4R,5S)-10-(benzyloxy)-9-methoxy-8,11-dimethyl-3-(thiazol-5-ylmethyl)-1,2,3,4,5,6-hexahydro-1,5-epiminobenzo[d]azocine-4-carbonitrile [**3e** (**DH_25**)]*

To a solution of lactam **2e** (68.6 mg, 153 μmol) in THF (3.4 mL) at 0 °C was slowly added $LiAlH_2(OEt)_2$ (1.0 mol/L in CH_2Cl_2, 1.83 mL, 1.83 mmol, 12 equiv.) over 10 min. The reaction mixture was stirred at 0 °C for 3 h. The reaction mixture was quenched with AcOH (185 μL, 3.20 mmol, 21 equiv.), followed by the addition of KCN (62.1 mg, 916 μmol, 6.0 equiv.) in H_2O (250 μL), and stirring was continued for 18 h at 25 °C. The reaction mixture was neutralized with 5% $NaHCO_3$ solution and diluted with saturated Rochell's salt aq., and the mixture was stirred for 1 h. The reaction mixture was extracted with $CHCl_3$ (3 × 80 mL). The combined extracts were washed with brine (80 mL), dried over Na_2SO_4, and concentrated in vacuo to give a residue. The residue was purified by SiO_2 flash column chromatography ($CHCl_3$:MeOH = 19:1) to afford compound **3c** (**DH_25**, 61.8 mg, 88%) as a colorless amorphous.

3e (**DH_25**): $[\alpha]_D^{24}$ −20.1 (*c* 0.19, $CHCl_3$). ^{1}H-NMR (500 MHz, $CDCl_3$) δ: 8.64 (1H, s), 7.57 (1H, s), 7.38-7.29 (5H, m), 6.68 (1H, s), 5.05 (1H, d, *J* = 11.5 Hz), 4.96 (1H, d, *J* = 11.5 Hz), 3.89 (1H, s), 3.83 (3H, s), 3.78-3.70 (3H, m), 3.23 (1H d, *J* = 8.2 Hz), 3.02 (1H, dd, *J* = 17.6, 8.2 Hz), 2.85 (1H, dd, *J* = 10.5, 3.0 Hz), 2.53 (1H, d, *J* = 10.5 Hz), 2.35 (1H, d, *J* = 17.6 Hz), 2.31 (3H, s), 2.12 (3H, s). ^{13}C-NMR (125 MHz, $CDCl_3$) δ: 153.8, 148.9, 148.3, 141.7, 137.5, 135.7, 130.3, 129.9, 128.6, 128.5, 128.2, 126.0, 124.4, 116.1, 74.5, 60.1, 58.9, 55.3, 53.6, 52.7, 51.3, 41.2, 24.9, 15.9. IR (KBr) cm^{-1}: 2936, 2825, 1414, 1321, 1229, 1160, 1061, 1029, 874, 754, 700, 603, 438, 427, 412, 405. EI-MS *m*/*z* (%): 460 (M^+, 2), 295 (25), 294 (100), 243 (11), 204 (20), 203 (22). HR-EI-MS *m*/*z*: 460.1933 (M^+, calcd for $C_{26}H_{28}N_4O_2S$, 460.1934).

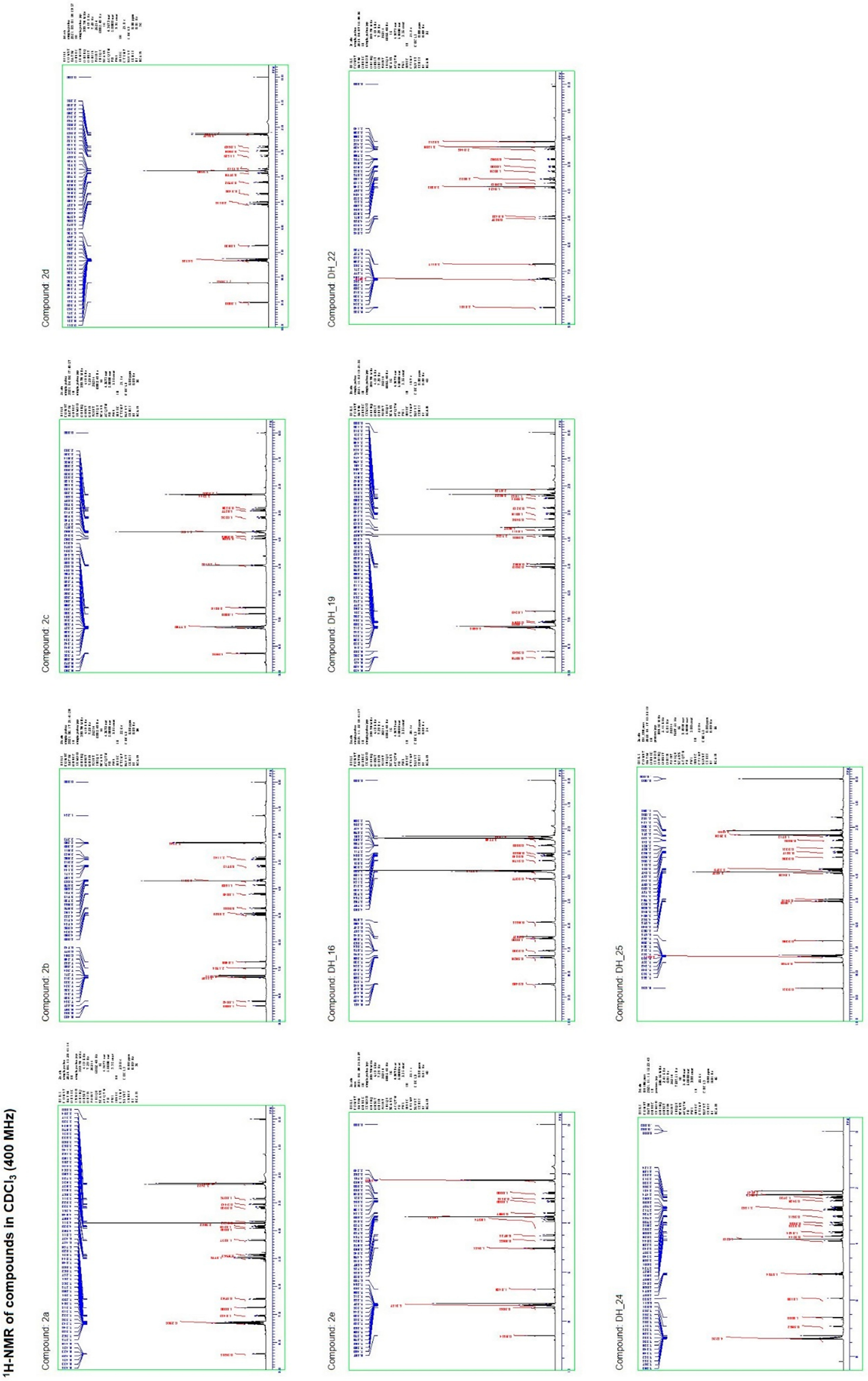

Figure S4. ^{1}H-NMR of derivatives of RT right-half analog (DH_16, DH_19, DH_22, DH_24, and DH_25) in $CDCl_3$ (400 MHz)

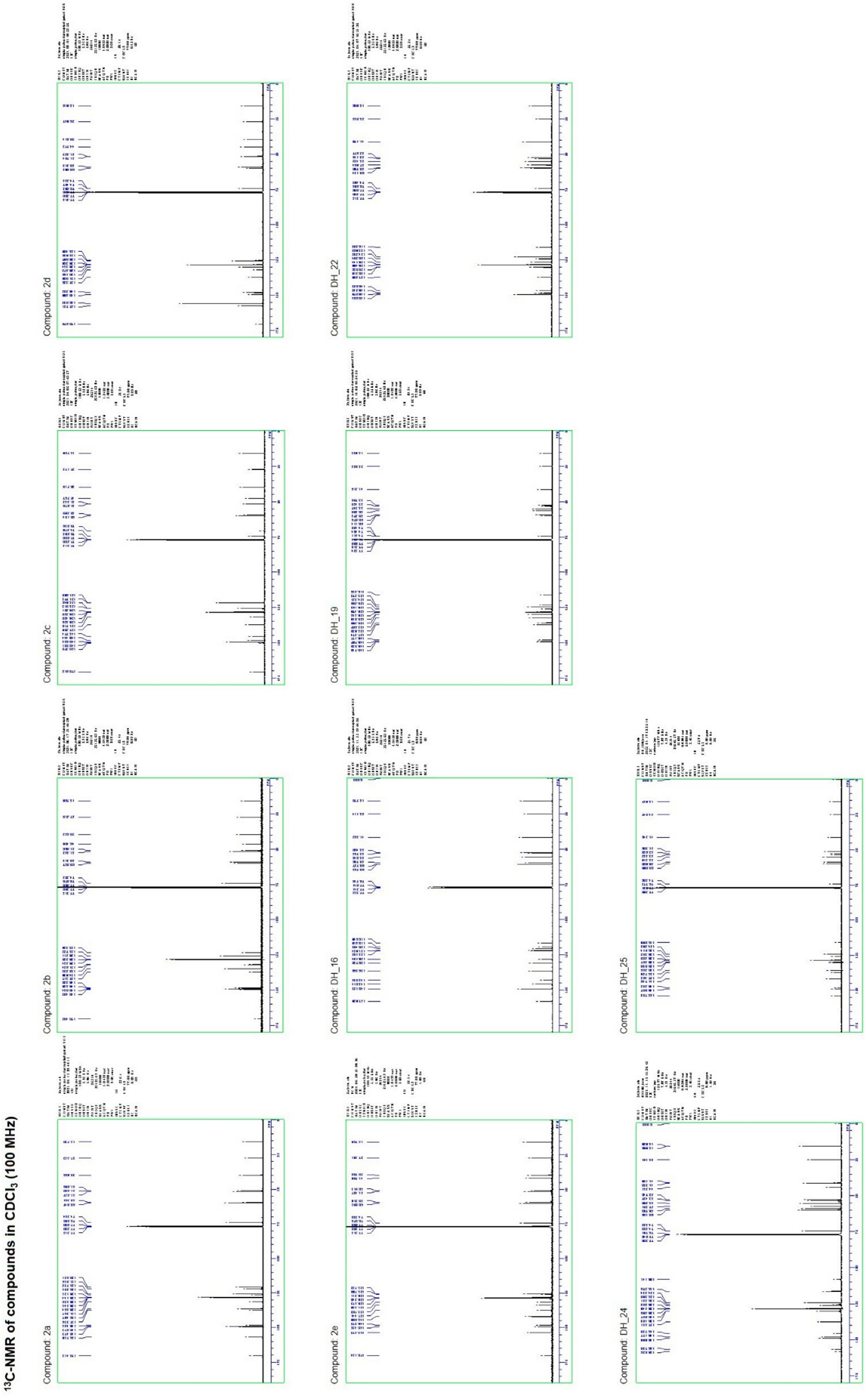

Figure S5. ^{13}C-NMR of derivatives of RT right-half analog (DH_16, DH_19, DH_22, DH_24, and DH_25) in $CDCl_3$ (100 MHz)

Reference

1. Matsubara, T.; Yokoya, M.; Sirimangkalakitti, N.; Saito, N. Asymmetric Synthesis and Cytotoxicity Evaluation of Right-Half Models of Antitumor Renieramycin Marine Natural Products. *Marine drugs* **2018**, *17*, 3, doi:10.3390/md17010003.
