# Supplementary figures and images for: Novel Synthetic Derivative of Renieramycin T Right-Half Analog Induces Apoptosis and Inhibits Cancer Stem Cells via Targeting the Akt Signal in Lung Cancer Cells
Source: Int J Mol Sci. 2023 Mar 10;24(6):5345. doi: 10.3390/ijms24065345 (PMC10049402; doi:10.3390/ijms24065345)

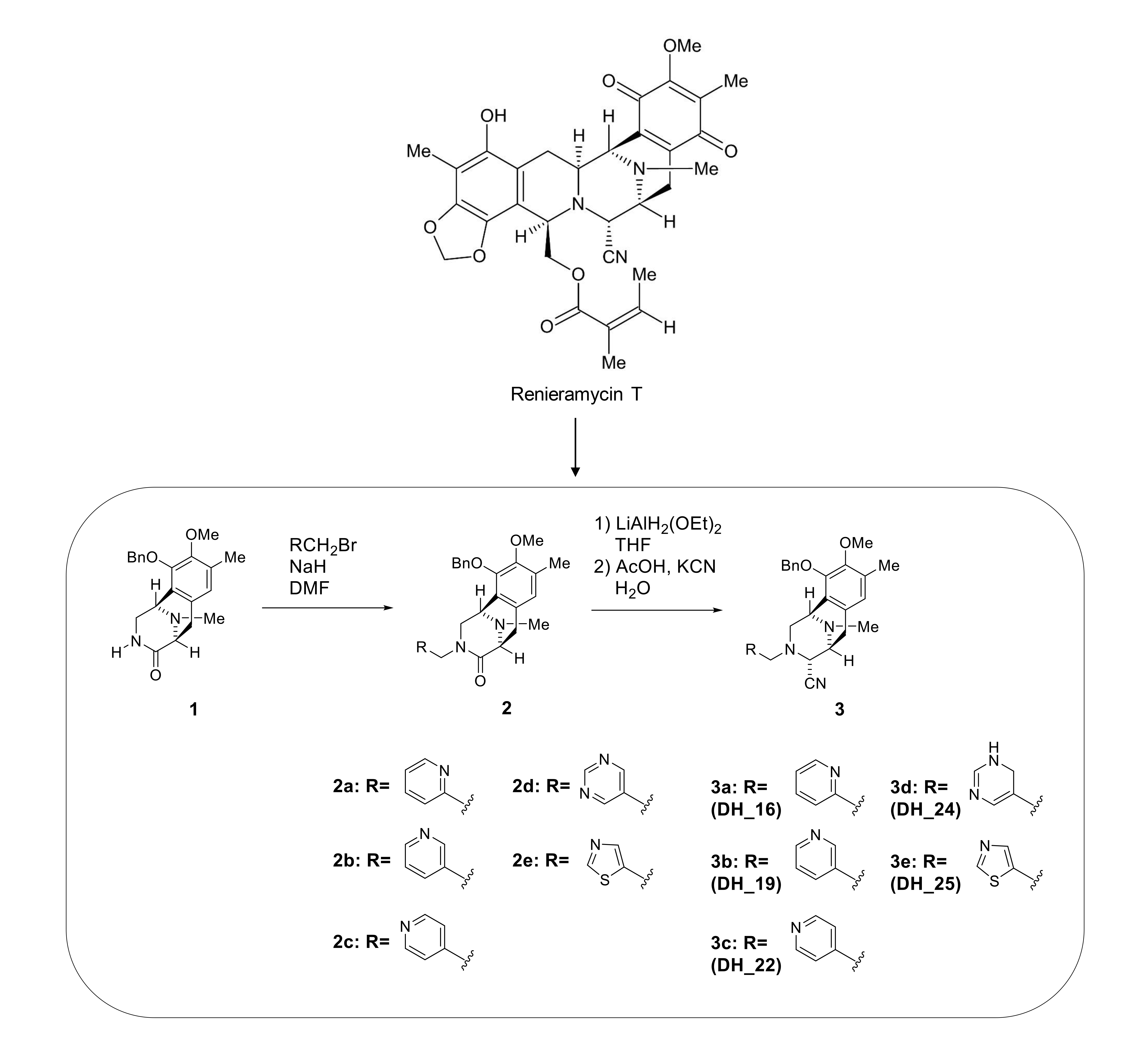

Supplement: Supplementary file 1 [file ijms-24-05345-s001.zip › Figure S3.jpg]

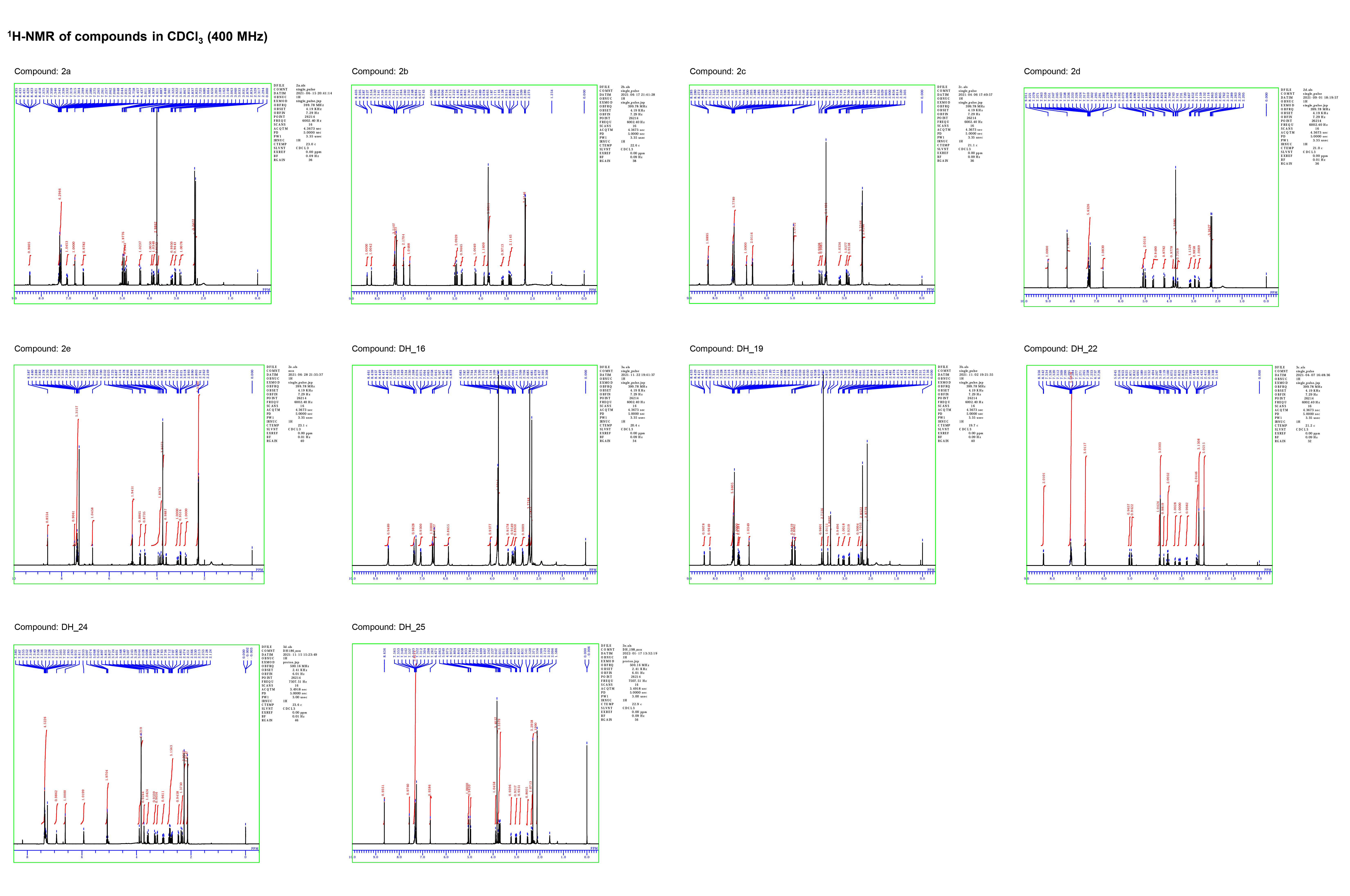

Supplement: Supplementary file 1 [file ijms-24-05345-s001.zip › Figure S4.jpg]

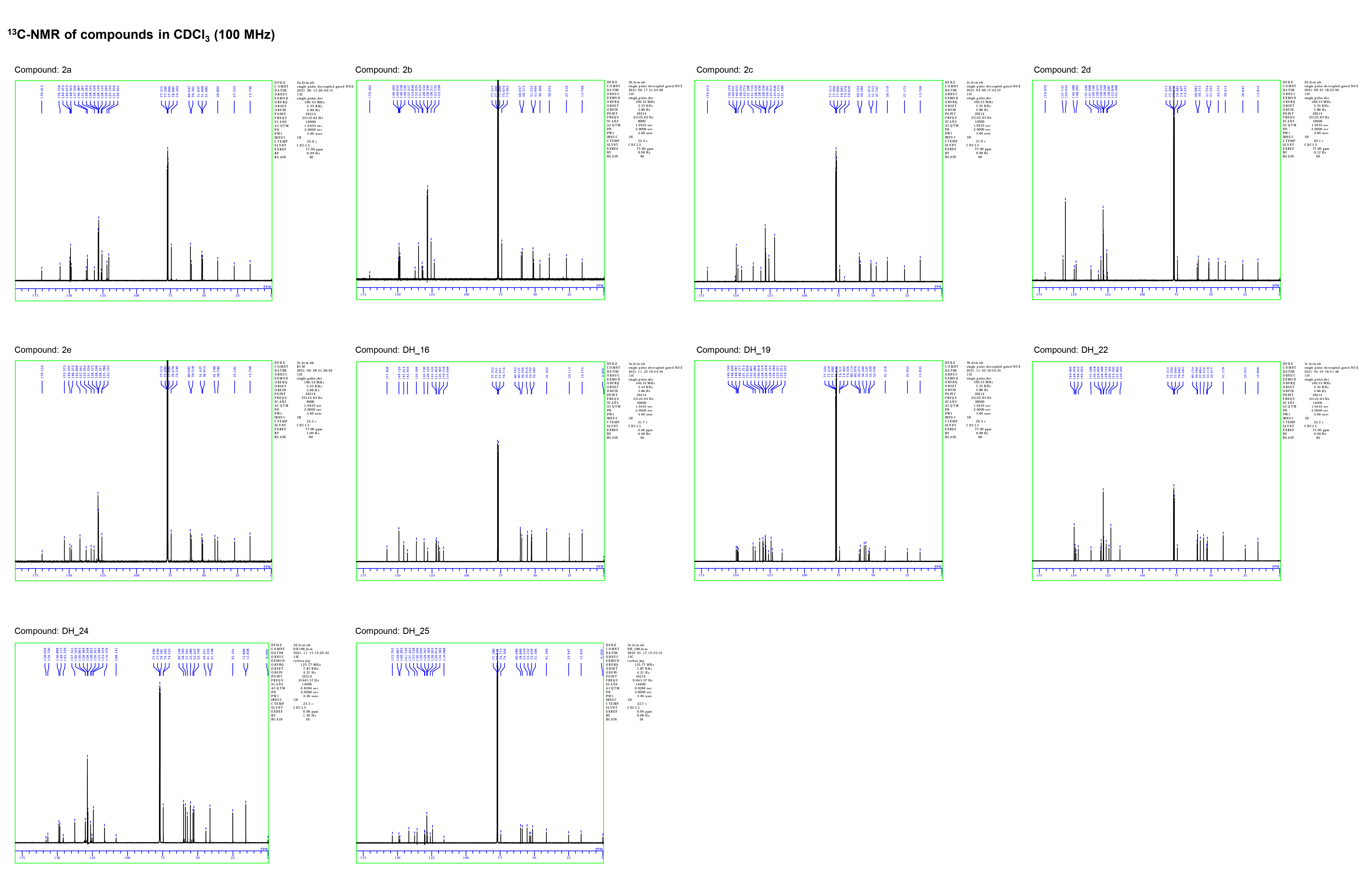

Supplement: Supplementary file 1 [file ijms-24-05345-s001.zip › Figure S5.jpg]

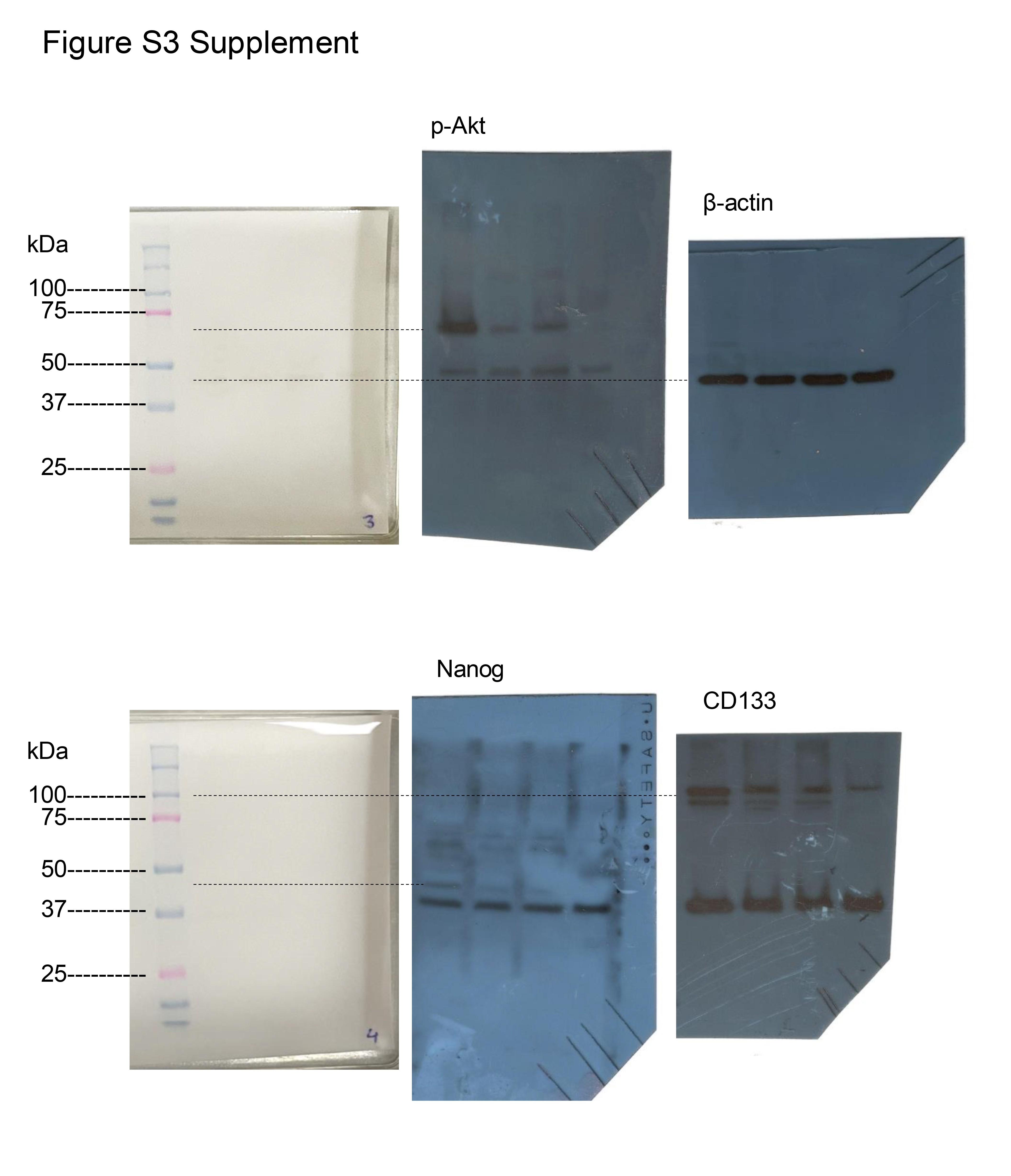

Supplement: Supplementary file 1 [file ijms-24-05345-s001.zip › Figure S8.jpg]
